# A Multi-omics approach to identify and validate shared genetic architecture in rheumatoid arthritis, multiple sclerosis, and type 1 diabetes: integrating GWAS, GEO, MSigDB, and scRNA-seq data

**DOI:** 10.1007/s10142-025-01598-x

**Published:** 2025-04-21

**Authors:** Tailin Wang, Qian He, Kei Hang Katie Chan

**Affiliations:** 1 Department of Biomedical Sciences, City University of Hong Kong, Kowloon, Hong Kong SAR China; 2 Department of Electrical Engineering, City University of Hong Kong, Kowloon, Hong Kong SAR China; 3 Department of Epidemiology, Centre for Global Cardiometabolic Health, Brown University, Rhode Island, USA; 4 City University of Hong Kong, Room 1A-313, 3/F, Block 1, To Yuen Building, 31 To Yuen Street, Kowloon Tong, Kowloon, HKSAR China

**Keywords:** *ROMO1*, Rheumatoid arthritis, Multiple sclerosis, Type 1 diabetes, ROS

## Abstract

The notable comorbidity among autoimmune diseases underscores their shared genetic underpinnings, particularly evident in rheumatoid arthritis (RA), type 1 diabetes (T1D), and multiple sclerosis (MS). However, the exact components and mechanisms of this shared genetic structure remain poorly understood. Here we show that *ROMO1* is a key shared genetic component among RA, MS, and T1D. Using differential gene expression (DGE) and LASSO regression analyses of bulk RNA-seq data from whole blood tissues, we identified *ROMO1* as a potential shared genetic factor. A multi-sample analysis with external Gene Expression Omnibus (GEO) data revealed *ROMO1’s* consistent association with immune cell patterns across tissues in all three diseases. Single-gene Gene Set Enrichment Analysis (GSEA) suggested *ROMO1’s* involvement in the reactive oxygen species (ROS) pathway, which was further substantiated by conjoint analysis with 256 ROS pathway-related genes(ROSGs) from Molecular Signatures Database (MSigDB). Single-gene Receiver Operating Characteristic (ROC) analysis highlighted *ROMO1’s* potential as a disease biomarker. Single-cell RNA sequencing (scRNA-seq) analysis showed significantly altered *ROMO1* expression in monocytes and other immune cells compared to healthy control (HC). Immune infiltration analysis revealed *ROMO1’s* significant association with monocytes across all three diseases. Furthermore, two-sample Mendelian randomization (MR) analysis using genome-wide association studies (GWAS) data demonstrated that *ROMO1* could regulate epitopes on monocytes, potentially lowering autoimmune disease risk. Our findings clarify the importance of *ROMO1* in the shared genetic architecture of RA, MS, and T1D, and its underlying mechanism in disease development.

**Supplementary Information:**

The online version contains supplementary material available at 10.1007/s10142-025-01598-x.

## Clinical trial number

Not applicable (this study is based on bioinformatics analysis and does not involve a clinical trial).

## Background

Autoimmune diseases such as type 1 diabetes (T1D), rheumatoid arthritis (RA), and multiple sclerosis (MS) have long posed significant challenges in medical science (Fugger et al. 2020; Barati et al. 2023). These diseases not only severely affect patients’ quality of life but can also lead to severe complications and even death (Thomas et al. 2010; Mitratza et al. 2021; Miller 2023). According to data from studies conducted worldwide, the annual incidence and prevalence of autoimmune diseases have increased by 19.1% and 12.5%, respectively (Miller 2023). In the UK, a large-scale study based on Clinical Practice Research Datalink (CPRD) data found that the overall incidence of autoimmune diseases increased by 4% (IRR 1.04) between 2000 and 2019 (Conrad et al. 2023). The study also revealed that autoimmune diseases affect around 10.2% of the UK population, with a significantly higher prevalence in women (13.1%) than men (7.4%).

However, the challenges of autoimmune diseases are not limited to their increasing prevalence. These diseases also exhibit significant comorbidity, meaning that patients diagnosed with one autoimmune disease are more likely to develop another (Cojocaru et al. 2010; Sarfaraz and Anis 2020). This pattern of comorbidity is particularly evident in patients with RA, MS, and T1D, prompting researchers to consider the possibility of shared genetic foundations and mechanisms underlying these diseases (Almeida et al. 2022; Pitzalis et al. 2008). For instance, studies have found that the prevalence of RA and T1D is relatively high among MS patients, while T1D patients are at a greater risk of developing RA (Lernmark 2002; Hojjati et al. 2016; Liao et al. 2009). More intriguingly, certain drugs used to treat RA have shown potential in inhibiting the progression of T1D (Waibel et al. 2023). The comorbidity of autoimmune diseases further exacerbates the complexity of diagnosis, treatment, and management (Nociti and Romozzi 2022).

In recent years, the rapid development of genomics and metabolomics has brought breakthrough progress to the study of autoimmune diseases. Among these advancements, mitochondrial dysfunction has increasingly become a focus of research. The latest genomic and metabolomic studies have revealed an exciting discovery: the dysregulation of reactive oxygen species (ROS) may play a crucial role in the pathogenesis of multiple autoimmune diseases (Hanaford and Johnson 2022). These studies suggest that certain genetic variations might lead to abnormal ROS production by affecting mitochondrial function, indicating the potential existence of ROS pathway-related shared genetic architecture (Miyata et al. 2020; Ghasemi et al. 2020; Kim et al. 2017). However, the exact components of this shared genetic structure remain to be fully elucidated, while our understanding of their precise mechanisms of action and regulatory roles in disease progression are still very limited. Therefore, it becomes particularly important to thoroughly analyze the component of the shared genetic architecture related to ROS and their mechanisms of action in autoimmune diseases.

In this study, we employed a multi-omics approach, integrating genome-wide association studies (GWAS), Gene Expression Omnibus (GEO), Molecular Signatures Database (MSigDB), and single-cell RNA sequencing (scRNA-seq) data to identify and validate shared genetic architecture in RA, MS, and T1D. Our aim is to provide new insights into these complex autoimmune diseases, potentially laying the groundwork for future precision medicine and personalized treatment strategies.

## Materials and methods

### Over design

**Fig. 1:**
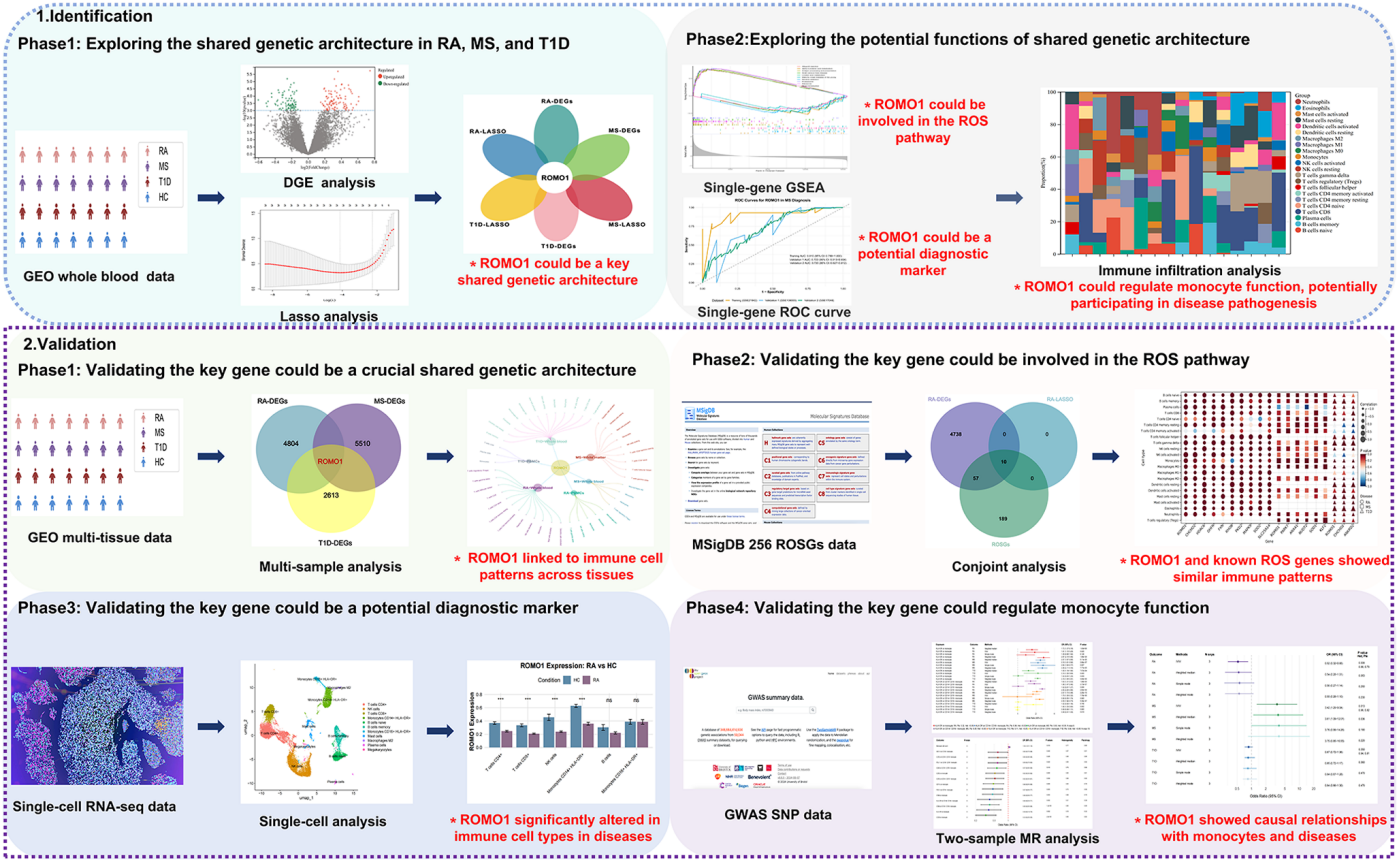
The flow chart of the research

In this study, we adopted a multidimensional and comprehensive research strategy integrating multiple datasets to identify and validate the key shared genetic architecture in RA, MS, and T1D (Fig. 1). In the identification phase, we analyzed bulk RNA-seq data from whole blood tissues in GEO (RA: GSE205962; MS: GSE17048; T1D: GSE44314), performing differential gene expression (DGE) and Least Absolute Shrinkage and Selection Operator (LASSO) regression to identify the key shared genes. Subsequently, we explored gene functions through single-gene Gene Set Enrichment Analysis (GSEA), Receiver Operating Characteristic (ROC) curve analyses, and immune infiltration analyses. To validate the key findings of the identification phase, we performed multi-sample analysis using external GEO data, conducted conjoint analysis with 256 ROS pathway-related genes (ROSGs) from the MSigDB, carried out single-cell analysis using scRNA-seq data, and performed two-sample Mendelian randomization (MR) analysis using GWAS data.

### Data collection and processing

All bulk RNA-seq datasets were collected from the GEO database (http://www.ncbi.nih.gov/geo). Initial data processing was conducted using GEO2R, an interactive web tool provided by GEO for comparing gene expression across different groups of samples. We utilized GEO2R for data retrieval and preliminary analysis, which encompassed the identification of differentially expressed genes (DEGs) and the generation of log2-transformed expression values. GEO2R automatically manages the conversion of probe identifiers to gene symbols based on the annotation information specific to each platform. For subsequent processing and in-depth analysis, we exported the GEO2R results and imported them into R statistical software. Additionally, GWAS data were obtained from the GWAS database (https://gwas.mrcieu.ac.uk/). 256 ROSGs were obtained from the MSigDB (https://www.gsea-msigdb.org/gsea/msigdb). scRNA-seq data were also downloaded from GEO database and processed using standard quality control, normalization, dimensionality reduction, and cell type annotation procedures.

### Identification stage: exploring the shared genetic architecture and its potential functions in RA, MS, and T1D

In this section, we aimed to explore the shared genetic architecture and its potential functions in RA, MS, and T1D using bulk RNA-seq data from whole blood tissues. Our analysis was conducted in two phases. Phase 1 focused on exploring the shared genetic architecture in RA, MS, and T1D, which included DGE analysis and LASSO analysis. Phase 2 aimed at exploring the potential function of this shared genetic architecture, comprising single-gene GSEA, single-gene ROC analysis, and immune infiltration analysis.

### DGE analysis

To identify the shared DEGs among RA, MS, and T1D from the whole blood samples, we performed the DGE analysis. We obtained the bulk RNA-seq data from the GEO database and the accession numbers for these diseases were GSE205962 for RA, GSE17048 for MS, and GSE44314 for T1D (Table 1). After obtaining the datasets from the GEO database, we implemented the “limma” R package to identify RA − related, MS-related and T1D-related DEGs in patients and the “ggplot2” R package to draw the volcano plot of the DEGs (Guo et al. 2019; Li etal. 2022a, b). We used an empirical Bayes method with a linear model fit. The Benjamini & Hochberg (False discovery rate) method was applied to adjust the P-values for multiple testing. The cut-off values were set to adjusted P-value < 0.05 for statistical significance. The “VennDiagram” R package was employed to identify shared DEGs among the three diseases (Yan et al. 2022; O’Brien 2018). We then compared these shared DEGs with those in healthy controls (HC) using Wilcoxon rank-sum tests, with P-value < 0.05 as the significance threshold.

**Table 1:** Detailed information of included GEO data for DGE analysis

Disease	GEO ID	Platform	Tissue	Case	Control	Total
RA	GSE205962	GPL16043	Whole blood	16	4	20
MS	GSE17048	GPL6947	Whole blood	99	45	144
T1D	GSE44314	GPL6480	Whole blood	10	6	16

### LASSO regression analysis

To identify the key shared genes among RA, MS, and T1D, we analyzed the shared DEGs associated with the three diseases using the LASSO analysis, executed through the “glmnet” R package (Liu et al. 2023). We used 10-fold cross-validation to select the optimal lambda value. By adjusting the penalty parameter λ, LASSO reduces the complexity of the model, shrinking some coefficients towards zero to select the most relevant features. We selected the largest lambda value within one standard error of the minimum mean cross-validated error (lambda.1se) to obtain a more parsimonious model. The coefficients of the selected genes were extracted based on this lambda value. To evaluate the accuracy of these key genes, we used the “survivalROC” R package to compute their ROC values (Zheng et al. 2021). The steps of feature selection, model optimization and ROC value were all aimed at controlling false positives. Subsequently, we intersected these key genes and the overlap of RA, MS, and T1D key genes were visualized using the “VennDiagram” R package (Chen et al. 2022).

### Single-gene GSEA

To identify the potential biological functions of key shared genetic architecture, we calculated the Pearson correlation coefficients between gene and all other genes implicated in the three diseases. Then, we utilized the “clusterProfiler” R package’s “GSEA” function to perform single-gene GSEA, along with Gene Ontology (GO) and Kyoto Encyclopedia of Genes and Genomes (KEGG) pathway analyses, based on the ranked correlation coefficients, to determine whether the predefined gene sets were significantly enriched (Zhan et al. 2022). GSEA was performed with 10, 000 permutations, setting the minimum and maximum gene set sizes to 10 and 200. The significance of the enriched pathways was determined based on the adjusted P-value provided by the GSEA algorithm, with adjusted P-value < 0.05 as the significance threshold. The enrichment results were visualized using the “gseaplot2” function from the “enrichplot” R package, highlighting significantly positively and negatively enriched pathways in each analysis category (Fisher et al. 2022). In this analysis, the 10, 000 permutation test, the minimum and maximum size of the gene set (10 and 200, respectively), and the adjusted P-value that came with the GSEA built-in algorithm were all used to control the false positive rate.

### Single-gene ROC curve analysis

We conducted single-gene ROC curve analysis using GEO datasets to evaluate key shared genetic architecture as potential diagnostic marker for RA, MS, and T1D. For each disease, we used one training set and two validation sets: RA (training: GSE56649, validation: GSE15573, GSE205962), MS (training: GSE21942, validation: GSE108000, GSE17048), and T1D (training: GSE44314, validation: GSE19273, GSE33440) (Supplementary Table 1). We used the “GEOquery” R package to download data, the “limma” R package for normalization, the “pROC” R package to construct ROC curves and calculate Area Under the Curve (AUC) values, and the “ggplot2” R package for visualization (Guo et al. 2019; Li et al. 2022a, b; Zheng et al. 2021). For each disease, we first analyzed the training set to construct the ROC curve, calculate AUC, and determine the optimal threshold by maximizing the Youden index (sensitivity + specificity − 1). This optimal threshold was then applied to the two validation sets to assess generalizability. The steps of evaluating AUC, and independent datasets were all aimed at controlling the false-positive rate.

### Immune infiltration analysis

To identify the potential interactions between key shared genetic architecture and immune cells in the three autoimmune diseases, we performed the immune infiltration analysis. Firstly the CIBERSORT website (http://CIBERSORT.stanford.edu/) was employed to analyze gene expression data from the three diseases, quantifying the relative proportions of 22 immune cell subsets (Yu et al. 2021). The analysis was performed using the default settings of CIBERSORTx, including the LM22 signature matrix (22 immune cell types) and 100 permutations. The false-positive rate was controlled by randomly disrupting the sample labels and recalculating the proportion of immune cells. Then we compared the immune cell infiltration between the disease group and HC using non-parametric tests (Wilcoxon rank-sum test). The significance threshold was set at P-value < 0.05 and the results were visualized using box plots with the “ggpubr” R package (Xiong et al. 2023). To illustrate the correlation between key gene and immune cell subsets, Pearson correlation coefficients were used for the correlation analysis between key gene and immune cell subsets, with a significance threshold of P-value < 0.05, and heatmaps were generated with the “pheatmap” R package (Lian et al. 2024; Hu and Chen 2022).

### Validation stage: validating the key findings and its potential functions in RA, MS, and T1D

Based on the key findings from the identification phase, which explored the shared genetic architecture and potential biological mechanisms in RA, MS, and T1D, we formulated several hypotheses regarding a key shared gene. These hypotheses proposed that this gene could be a crucial shared genetic architecture, potentially involved in the ROS pathway, serve as a diagnostic marker, and regulate monocyte function in these autoimmune diseases. In this section, we aimed to validate these hypotheses through four distinct phases.

### Multi-sample analysis

To validate that key gene could be a crucial shared genetic architecture in RA, MS, and T1D, we performed multi-sample analysis using GEO datasets from different tissues for each disease. Firstly, we analyzed the data from three peripheral blood cell tissues (RA: GSE56649, MS: GSE21942, T1D: GSE110914) from the GEO database to perform the DGE analysis (Supplementary Table 2) (Rosati et al. 2024). Then we intersected the DEGs of these three diseases to isolate the shared DEGs. And we further selected peripheral blood mononuclear cells (PBMCs) data from RA (GSE15573) and T1D (GSE193273) as well as brain tissue data from MS (GSE108000) to perform the immune infiltration analysis (Supplementary Table 2) (Tyagi et al. 2023; Severin et al. 2016). Finally, we examined the correlation between key gene expression and immune infiltration across different tissues in RA, MS, and T1D to determine whether key gene was consistently associated with various immune cells in diverse disease contexts. The R packages and parameter settings for DGE analysis and immune infiltration analysis remained consistent with our previously described methods, employing “limma” for DGE analysis and “CIBERSORT” for immune cell deconvolution (Guo et al. 2019; Li et al. 2022a, b; Yu et al. 2021).

### Conjoint analysis

To validate the relationships between the key gene and ROS pathway in RA, MS, and T1D, we downloaded the 256 ROSGs data from the MSigDB and conducted a conjoint analysis (Supplementary Table 2) (Cen et al. 2023). We first performed DGE analyses on three autoimmune diseases (RA, GSE56649; MS, GSE17048; T1D, GSE44314) (Supplementary Table 4) (Guo et al. 2019). Following the identification of DEGs for each disease, we screened for overlapping genes between these DEGs and the set of 256 ROSGs. For the shared genes identified s with a count greater than 10, we performed Lasso regression analysis (Chintalapudi et al. 2022). Then these key genes and the overlap was visualized using the “VennDiagram” R package (Chen et al. 2022). Finally, we performed the immune infiltration analysis for each disease, specifically focusing on the shared genes identified in the conjoint analysis, and correlated their expression levels with the relative proportions of various immune cell subsets. The R packages and parameter settings for DGE analysis, Lasso regression analysis and immune infiltration analysis remained consistent with our previously described methods, employing “limma” for DGE analysis, “glmnet” R package for Lasso regression analysis and “CIBERSORT” for immune cell deconvolution (Li et al. 2022a, b; Liu et al. 2023; Yu et al. 2021).

### Single-cell analysis

To validate the key gene could be a potential diagnostic marker in RA, MS, and T1D, we obtained single-cell data for RA, MS, T1D, and HC for single-cell analysis (Supplementary Table 5). We conducted the single-cell analyses separately for each of the four groups (RA, MS, T1D, and HC) using the “Seurat” R package (Liu et al. 2024). During data processing, we first applied strict threshold screening conditions (200 < nFeature_RNA < 2500, percent.mt < 5%) to remove low quality cells. Subsequently, we performed cell counting, total number of reads, and in-depth analysis of the data. For datasets with more than 10, 000 cells, we further implemented a double cell removal step to eliminate potential doublets. The elimination of low quality cells and double cells helped to minimize false positives due to technical variability and batch effects. Next, we normalized the data using the LogNormalize method (scale.factor = 10, 000) and selected high variant genes using the variance stable transformation (vst) method (*n* = 2000). After dimensionality reduction, we performed cell clustering and employed t-distributed Stochastic Neighbor Embedding (t-SNE) for visualization (Supplementary Table 6) (Liao et al. 2024). We then calculated the average expression levels of key gene across cell clusters for each sample. Subsequently, we compared the key gene expression levels in each autoimmune disease group with those in the HC group using two-sample t-tests, performed with the “stats” R package. The significance threshold was set at P-value < 0.05. This comprehensive analysis included generating four visualization plots for each group: t-SNE clustering plots, key gene expression feature plots, bar plots of key gene average expression levels across cell clusters, and comparison plots of key gene differential expression between disease groups and HC in various cell clusters.

### Two-sample MR analysis

To validate the exact associations among three autoimmune diseases, monocytes and the key gene, we performed a two-sample MR analysis using the “TwoSampleMR” R package (Xu et al. 2023a, b; Nica and Dermitzakis 2013). The analysis was conducted in three sequential steps. We first analysed the causal relationship between the key gene expression (exposure) and monocytes (outcome), using GWAS data for 30 monocyte-related datasets, including phenotypes and cell count, and eQTL data (eqtl-a-ENSG00000125995) for the key gene (Supplementary Table 7). We then investigated the causal relationship between monocyte-related datasets (exposure), including phenotypes and cell count, and the three autoimmune diseases (outcome). For this, we used GWAS data for the 14 monocytes (identified as significant in the first step) and GWAS data for RA (finn-b-RHEUMA_SEROPOS_OTH), MS (finn-b-G6_MS), and T1D (ebi-a-GCST90014023) (Supplementary Table 7). Finally, we analysed the causal association of the key gene expression (exposure) on the three autoimmune diseases (outcomes). Instrumental variables (IVs) were selected using stringent criteria to ensure the validity of our analysis.

In MR analysis, this study adopted a stratified screening strategy to ensure the rigor of causal inference. The specific implementation steps were as follows: In the gene expression-disease association analysis (step 1 and step 3), the screening criteria for IVs were: genome-wide significance threshold *P* < 5 × 10^− 6^, linkage disequilibrium parameter r^2^ < 0.001, and physical distance window of 100 kb. For monocyte and autoimmune disease studies (step 2), the physical distance window was extended to 10, 000 kb to obtain a wider range of genetic variation information, taking into account the complexity of biological pathways.To mitigate weak instrument bias, we used the F-statistic to assess instrument strength, with a strict threshold of F > 20 (above the conventional F > 10) to ensure robustness. The core analysis employed the inverse-variance weighted (IVW) method, which performed intercept-free regression weighted by the reciprocal of outcome variances, with statistical significance defined as *P* < 0.05. Given IVW’s sensitivity to pleiotropic effects, a dual validation framework was established: Cochran’s Q test evaluated heterogeneity within the IVW framework, while MR-Egger regression’s intercept term (egger_intercept) detected directional pleiotropy, both utilizing *P* < 0.05 as significance thresholds (Hemani et al. 2018). This methodological design systematically identified potential pathways through which instrumental variables might influence outcomes beyond the exposure mechanism, thereby reinforcing causal inference reliability and implementing controls on false positive rates.

## Results

### *ROMO1* could be a crucial shared genetic architecture in RA, MS, and T1D

**Fig. 2:**
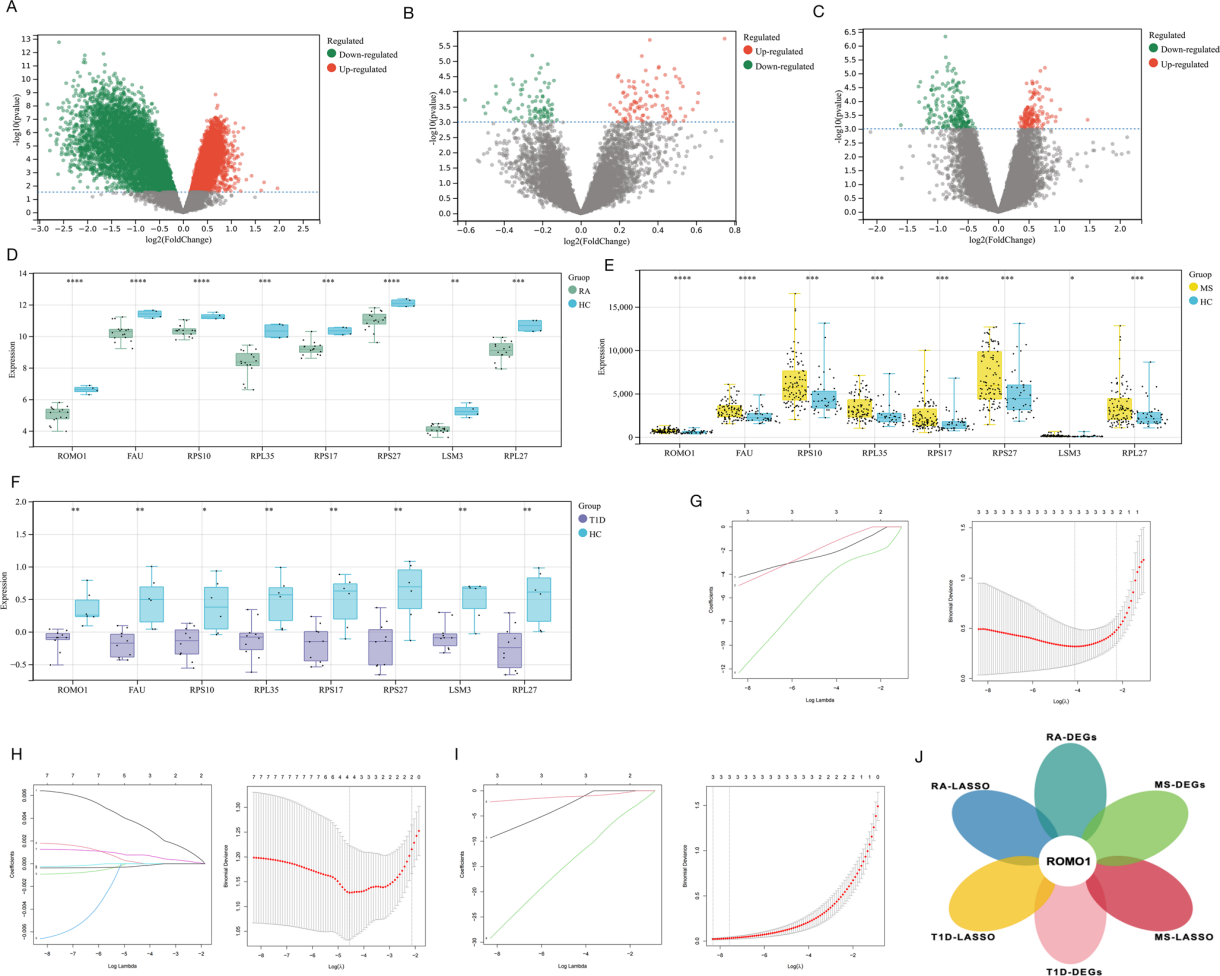
(**A**) The volcano plot displays the expression patterns of DEGs in RA.(**B**) The volcano plot displays the expression patterns of DEGs in MS. (**C**) The volcano plot displays the expression patterns of DEGs in T1D. (**D**) Comparison of the expresssion of 8 shared genes between RA and healthy controls(*P < 0.05, **P < 0.01, ***P < 0.001, ****P < 0.0001). (**E**) Comparison of the expresssion of 8 shared genes between MS and healthy controls(*P < 0.05, **P < 0.01, ***P < 0.001, ****P < 0.0001). (**F**) Comparison of the expresssion of 8 shared genes between T1D and healthy controls(*P < 0.05, **P < 0.01, ***P < 0.001, ****P < 0.0001). (**G**)Lasso regression model to identify candidate genes in RA. (**H**)Lasso regression model to identify candidate genes in MS. (**I**)Lasso regression model to identify candidate genes in T1D. (**J**)The Venn diagram displays the hub gene *ROMO1* among T1D, RA, and MS

We first conducted the DGE analysis for three autoimmune diseases: RA, MS, and T1D(Figs. 2A, B, C). Through cross-comparison, we identified 8 genes that were differentially expressed across all three diseases: *ROMO1*, * FAU*, * RPS10*, * RPL35*, * RPS17*, * RPS27*, * LSM3*, and *RPL27*(Supplementary Fig. 1). Further analysis of the expression patterns of these shared DEGs revealed that their expression levels were generally reduced in RA and T1D patients, while showing a trend of increased expression in MS patients(Fig. 2D, E, F). To further identify the most relevant key genes, we performed LASSO regression analysis for each disease separately(Fig. 2G, H, I). We selected the optimal λ values for each disease: for RA, λ was set to 0.010, indicating the 2 genes as as the candidate genes for RA (*ROMO1* and *LSM3*); for MS, λ was determined to be 0.012, identifying 2 genes for MS (*ROMO1* and *FAU*); for T1D, λ was set at 5.14 × 10^− 4^, identifying 3 genes for T1D (*ROMO1*, *LSM3* and *TPT1*). By cross-comparing the LASSO regression results, we found that *ROMO1* was the only gene selected in all three diseases(Fig. 2J). These findings strongly suggested that *ROMO1* may play a crucial role in these three autoimmune diseases.

### *ROMO1* could be involved in the ROS pathway in RA, MS, and T1D

**Fig. 3:**
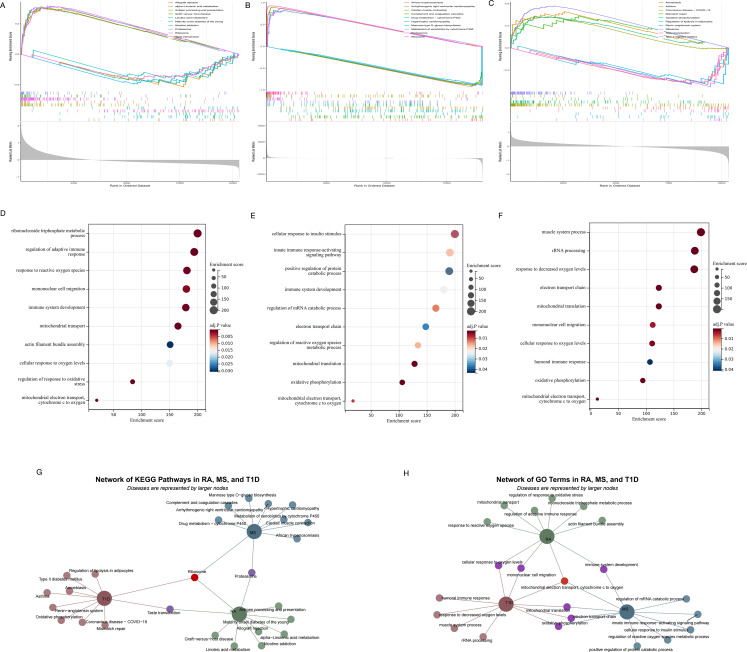
(**A**) KEGG enrichment analysis in RA. (**B**) KEGG enrichment analysis in MS. (**C**) KEGG enrichment analysis in T1D. (**D**) Go enrichment analysis in RA. (**E**) GO enrichment analysis in MS. (**F**) GO enrichment analysis in T1D. (**G**) The network of KEGG pathways in RA, MS and T1D. (**H**) The network of GO pathways in RA, MS and T1D

After identifying *ROMO1* as a key gene in three autoimmune diseases, we conducted a single gene GSEA. We examined the top five and bottom five KEGG signaling pathways (Fig. 3A, B, C) and GO terms (Fig. 3D, E, F) associated with these diseases. The analysis revealed that *ROMO1* was significantly enriched in the KEGG ribosome pathway across all three conditions (Fig. 3G). Furthermore, GO analysis indicated that *ROMO1* was negatively correlated with the electron transfer from cytochrome c to oxygen in the mitochondrial electron transport chain in these diseases (Fig. 3H). Considering that both ribosomal function and mitochondrial electron transport chain were closely related to cellular redox status, and previous studies had demonstrated a direct link between *ROMO1* and ROS production (Zhou et al. 2021), we inferred that *ROMO1* was likely involved in the ROS pathway in RA, MS, and T1D. At the same time, we also noted that there the three autoimmune diseases had differences in terms of enrichment pathways. RA showed unique functional enrichment in ribonucleoside triphosphate metabolism and regulation of adaptive immune response, which differed markedly from MS and T1D. In addition, T1D was mainly involved in myosystem-related processes, whereas MS showed specific regulation of proteolytic metabolic processes. These differences also provided important clues to understanding the pathomechanisms of these autoimmune diseases.

### *ROMO1* could regulate monocyte function, potentially participating in disease pathogenesis

**Fig. 4:**
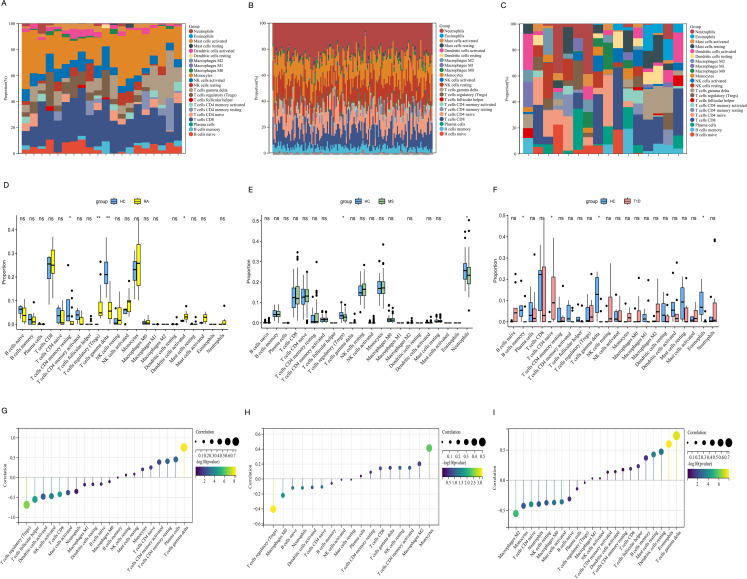
(**A**) Proportions of 22 immune cell types in RA. (**B**) Proportions of 22 immune cell types in MS. (**C**) Proportions of 22 immune cell types in T1D. (**D**) Comparison of the proportion of immune cell subsets between RA and healthy controls (*P < 0.05, **P < 0.01, ***P < 0.001, ns: not significant). (**E**) Comparison of the proportion of immune cell subsets between MS and healthy controls(*P < 0.05, **P < 0.01, ***P < 0.001, ns: not significant). (**F**) Comparison of the proportion of immune cell subsets between T1D and healthy controls(*P < 0.05, **P < 0.01, ***P < 0.001, ns: not significant). (**G**) Correlation between *ROMO1* and immune cell subsets of RA. (**H**) Correlation between *ROMO1* and immune cell subsets of MS.(**I**) Correlation between *ROMO1* and immune cell subsets of T1D

Using the CIBERSORT algorithm, we obtained the proportions of 22 different immune cell types in samples from three autoimmune diseases(Fig. 4A, B, C). The results of immune infiltration analysis, using non-parametric tests, also revealed significant differences in the immune cell composition between the HC and diseased groups across all the three diseases(Fig. 4D, E, F). Particularly the correlation heatmaps between *ROMO1* and the immune cells in three diseases further confirmed the critical role of *ROMO1* in the pathogenesis of these autoimmune disorders (Fig. 4G, H, I). *ROMO1* was found to be significantly associated with various types of immune cells in three diseases (Supplementary Fig. 2). Notably, consistent and significant associations with monocytes were found in all of these different disorders (RA, Cor = 0.20, *P* = 0.037; MS, Cor = 0.41, *P* = 3.51 × 10^− 3^; T1D, Cor=-0.43, *P* = 3.93 × 10^− 3^). Considering RA, MS and T1D as autoimmune diseases in which immune cells play a key role in the pathological process, and our analysis revealed that *ROMO1* showed significant correlation with monocytes in all three diseases. Therefore, we hypothesised that *ROMO1* could regulate monocyte function and thus participated in the pathogenesist of RA, MS, and T1D.

### *ROMO1* could be a potential diagnostic marker in RA, MS, and T1D

**Fig. 5:**
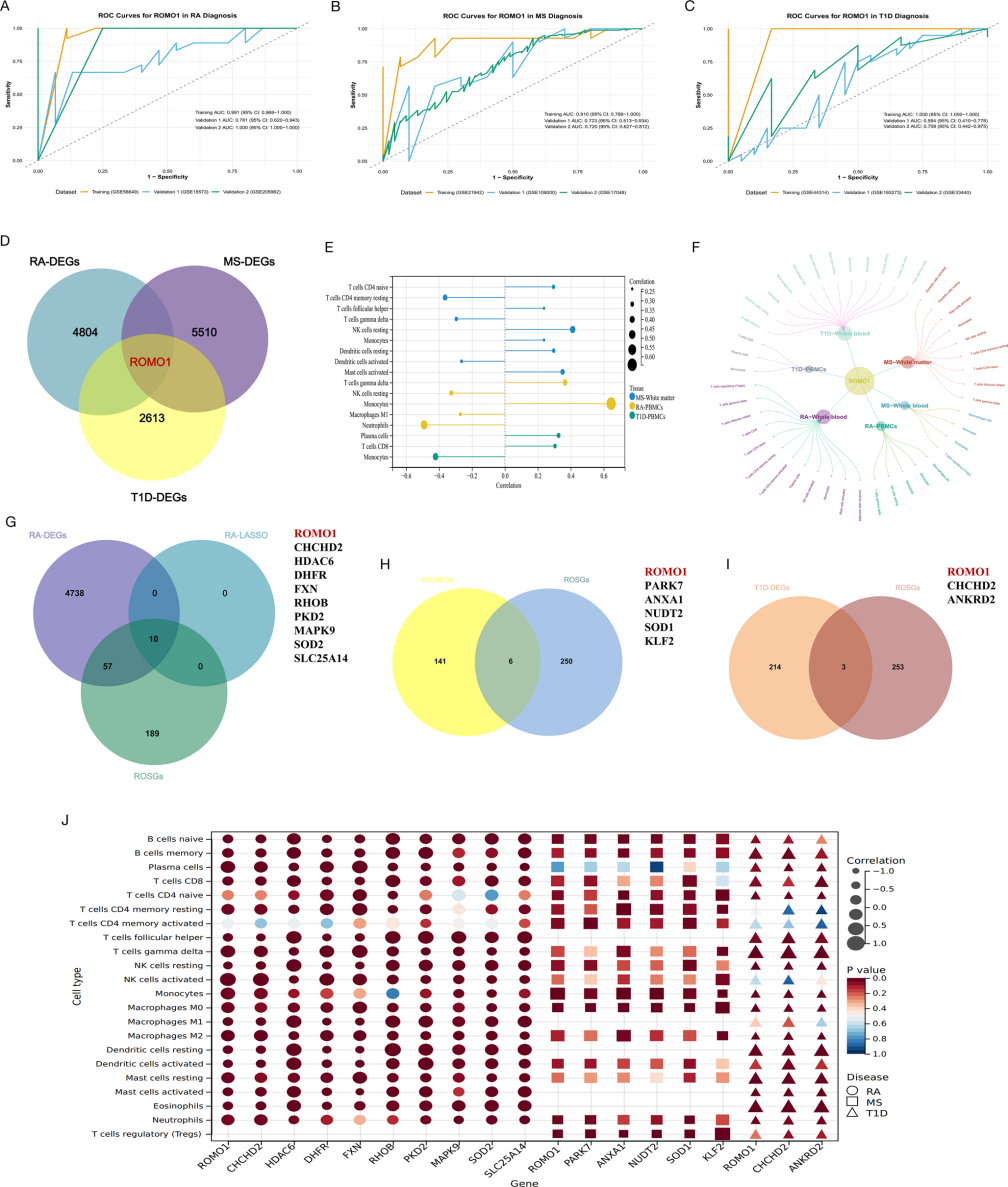
(**A**) The ROC curves for *ROMO1* in RA diagnosis. (**B**) The ROC curves for *ROMO1* in MS diagnosis. (**C**) The ROC curves for *ROMO1* in T1D diagnosis. (**D**)The Venn diagram displays the hub gene *ROMO1* among T1D, RA, and MS. (**E**) The significant correlations between *ROMO1* and RA, MS and T1D immune cell subsets. (**F**) Multi-tissue *ROMO1* correlation with immune subsets in RA, MS, T1D. (**G**) The Venn diagram displays the shared genes between RA and ROSGs. (**H**) The Venn diagram displays the shared genes between MS and ROSGs. (**I**) The Venn diagram displays the shared genes between T1D and ROSGs. (**J**) Correlation between shared genes and immune cell subsets of RA, MS and T1D

*ROMO1* demonstrated overall good diagnostic performance across three autoimmune diseases. In RA, *ROMO1* showed exceptional performance: the training set (GSE56649) achieved an AUC of 0.991 (95% CI: 0.968-1.000), validation set 1 (GSE15573) reached 0.781 (95% CI: 0.620–0.943), and validation set 2 (GSE205962) attained a perfect 1.000 (95% CI: 1.000–1.000), demonstrating high diagnostic accuracy and good generalizability(Fig. 5A). For MS, *ROMO1*’s diagnostic value was relatively consistent: the training set (GSE21942) had an AUC of 0.910 (95% CI: 0.789-1.000), while the two validation sets (GSE108000 and GSE17048) showed AUCs of 0.723 (95% CI: 0.513–0.934) and 0.720 (95% CI: 0.627–0.812) respectively, indicating moderate diagnostic accuracy for MS(Fig. 5B). In T1D, *ROMO1*’s performance varied across datasets: the training set (GSE44314) achieved an AUC of 1.000 (95% CI: 1.000–1.000), validation set 1 (GSE19273) reached 0.594 (95% CI: 0.410–0.778), and validation set 2 (GSE33440) showed an AUC of 0.708 (95% CI: 0.442–0.975), suggesting potential but variable diagnostic utility for T1D (Fig. 5C). We also performed the ROC analysis of *ROMO1* with these three diseases in supplementary results to compare the diagnostic ability of *ROMO1* with existing conventional biomarkers (Supplementary Fig. 8, 9, 10). Surprisingly, *ROMO1* outperformed typical biomarkers in the diagnosis of these diseases, showing higher diagnostic value. Taken together, these results indicate that *ROMO1* could be a potential diagnostic marker in RA, MS, and T1D, with varying degrees of performance across these autoimmune diseases.

### Validation of *ROMO1* could be a crucial shared genetic architecture in RA, MS, and T1D

We analyzed data from peripheral blood cell tissues of patients with RA, MS, and T1D, obtained from the GEO database. Our analysis consistently identified *ROMO1* as differentially expressed across these three autoimmune diseases(Fig. 5D). Further immune infiltration analysis revealed significant correlations between *ROMO1* and various immune cell types (Fig. 5E). Subsequently, by integrating all immunological findings, we observed consistent and significant associations between *ROMO1* and monocytes across different tissues in all three diseases (Fig. 5F). Collectively, these results validate that *ROMO1* could be a key component of the shared genetic architecture in RA, MS, and T1D.

### Validation of *ROMO1* could be involved in the ROS pathway in RA, MS, and T1D

After DGE and LSAAO regression analyses, we identified overlapping genes between DEGs and ROSGs in three autoimmune diseases. We found 10 overlapping genes in RA (including *ROMO1*, * CHCHD2*, * HDAC6*, * DHFR*, * FXN*, * RHOB*, * PKD2*, * MAPK9*, * SOD2*, * SLC25A14*), 6 in MS(including *ROMO1*, * PARK7*, * ANXA1*, * NUDT2*, * SOD1*, * KLF2*), and 3 in T1D (*ROMO1*, * CHCHD2*, * ANKRD2*). Notably, *ROMO1* was the only ROSG that overlapped in all three diseases (Fig. 5G, H, I). To further validated the above findings, we performed an immune infiltration analysis. The results showed that *ROMO1* and other key genes known to be involved in the ROS pathway (e.g., *CHCHD2*, * PARK7*, * ANXA1*, * NUDT2*, * SOD1*, and *ANKRD2*) exhibited a highly similar immune infiltration pattern in the three diseases(Fig. 5J). This remarkable similarity suggested that *ROMO1* may share similar functions and regulatory mechanisms with these known ROS pathway genes. These results strongly validated the critical role of *ROMO1* in the ROS pathway in RA, MS, and T1D.

### Validation of *ROMO1* could be a potential diagnostic marker in RA, MS, and T1D

**Fig. 6:**
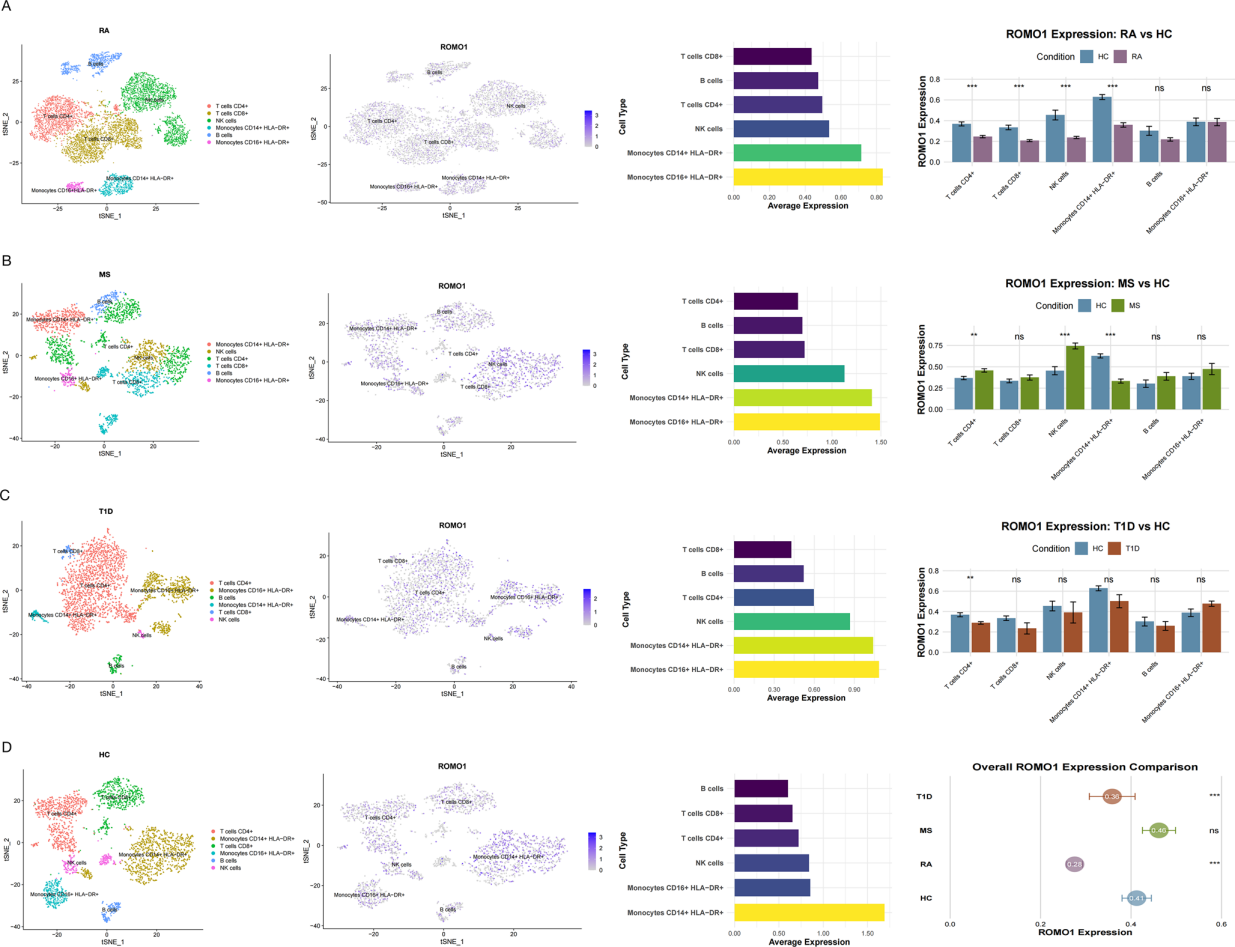
(**A**) The single cell analysis in RA(*P < 0.05, **P < 0.01, ***P < 0.001, ns: not significant). (**B**) The single cell analysis in MS(*P < 0.05, **P < 0.01, ***P < 0.001, ns: not significant). (**C**) The single cell analysis in T1D(*P < 0.05, **P < 0.01, ***P < 0.001, ns: not significant). (**D**) The single cell analysis in HC(*P < 0.05, **P < 0.01, ***P < 0.001, ns: not significant)

After quality control of the single-cell data, we obtained the following dataset-specific information: in the single-cell dataset for RA, the number of cells was 8, 564, the total number of reads was 36, 851, 544, and the depth of read per cell was 4, 304; in the single-cell dataset for MS, the number of cells was 2, 046, the total number of reads was 8, 709, 744 with a read depth of 4, 257 per cell; in the single-cell dataset for T1D, the number of cells was 3, 011 with 12, 827, 205 total reads and a read depth of 4, 260 per cell; in the single-cell dataset for the HC, the number of cells was 2, 961 with 15, 819, 466 total reads and the depth of read per cell was 5, 343. Subsequently, we performed cell classification analysis. The analysis identified six major immune cell populations (T cells CD4+, Monocytes CD16 + HLA-DR+, T cells CD8+, Monocytes CD14 + HLA-DR+, B cells, and NK cells) across four categories: RA, MS, T1D, and HC(Fig. 6A, B, C, D). *ROMO1* expression was detected in multiple cell types, with Monocytes CD14 + HLA-DR + and Monocytes CD16 + HLA-DR + showing the highest expression levels across all four groups. Compared to HC, RA samples exhibited significantly lower overall *ROMO1* expression (*P* < 0.001), with notable reductions in T cells CD4+ (*P* < 0.001), T cells CD8+, Monocytes CD14 + HLA-DR+ (*P* < 0.001), and NK cells (*P* < 0.001). While MS samples did not show significantly higher overall *ROMO1* expression compared to HC, we observed significant decreases in T cells CD4+ (*P* < 0.01), Monocytes CD14 + HLA-DR+ (*P* < 0.001), and NK cells (*P* < 0.001). In T1D samples, overall *ROMO1* expression was significantly lower than HC (*P* < 0.001), with a marked decrease in T cells CD4+ (*P* < 0.01). In conclusion, the consistency of *ROMO1*’s differential expression, particularly in key immune cell types such as CD4 + T cells and CD14 + HLA-DR + monocytes, further reinforces its potential as a robust biomarker.

### Validation of *ROMO1* could regulate monocyte function in RA, MS, and T1D

**Fig. 7:**
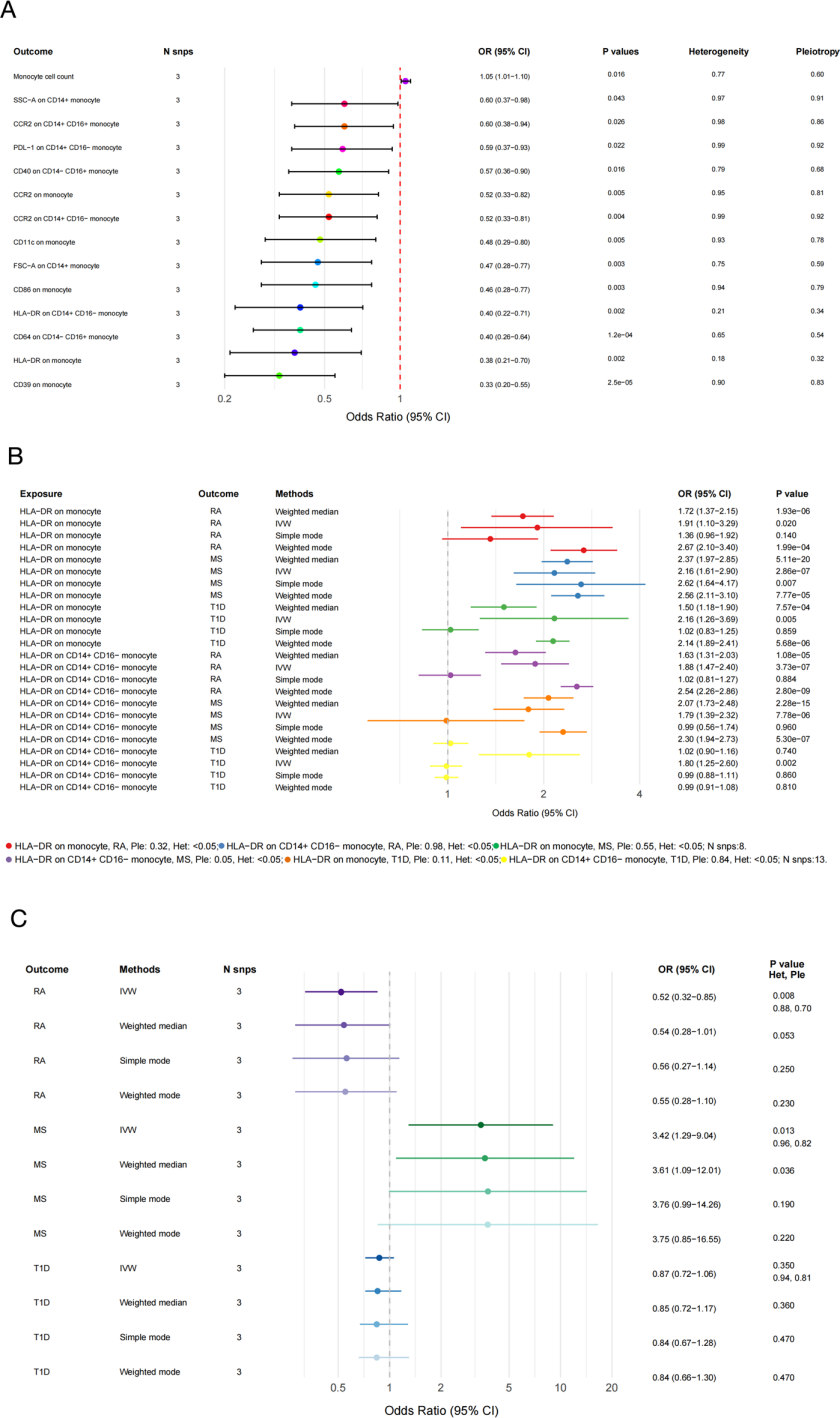
(**A**) The forest plot displays the effect estimates for the causal influence of upregulated *ROMO1* expression on the risk of monocytes. (**B**) The forest plot displays the effect estimates for the causal influence of monocytes on the risk of RA, MS and T1D. (**C**) The forest plot displays the effect estimates for the causal influence of upregulated *ROMO1* expression on the risk of RA, MS and T1D

After rigorous screening, we obtained three IVs for *ROMO1*. Subsequent MR analysis revealed significant associations between upregulated *ROMO1* expression and monocyte data across 14 types, with positive links to monocyte counts and negative links to 13 monocyte phenotypes (Fig. 7A). Then we used these 14 types of monocytes as exposure variables and the three autoimmune diseases as outcome variables. The results revealed that HLA-DR + monocytes, especially CD14 + monocytes, had OR greater than 1 for all three autoimmune diseases, suggesting these cells may increase disease risk (Fig. 7B). Finally, the two-sample MR analysis results showed that upregulated *ROMO1* expression was associated with an OR of 0.52 (95% CI: [0.32, 0.85], *P* = 0.0083) for RA, indicating that upregulated *ROMO1* expression might be a protective factor for RA(Fig. 7C). For MS, upregulated *ROMO1* expression correlated with an OR of 3.42 (95% CI: [1.29, 9.04], *P* = 0.013), suggesting that upregulated *ROMO1* might be a risk factor for MS. For T1D, the results for T1D were not significant, with an OR of 0.87 (95% CI: [0.72, 1.06], *P* = 0.35). These findings provided strong statistical evidence that *ROMO1* could regulate monocyte function, potentially participating in disease pathogenesis. We also assessed heterogeneity among IVs using Cochran’s Q test and tested for pleiotropy using the MR-Egger intercept method. The p-value of MR-Egger intercept method was greater than 0.05, indicating no significant pleiotropy. In the study of monocytes against three autoimmune diseases, although no pleiotropy was found, we observed some heterogeneity, so these results needed to be interpreted with caution. In summary, these findings provided strong statistical evidence that *ROMO1* could regulate monocyte function, potentially participating in disease pathogenesis.

### Supplementary methods and results

We also conducted several analyses to support these findings, including linkage disequilibrium score regression (LDSC) analysis, multi-trait analysis of GWAS (MTAG), cell communication analysis, bidirectional MR analysis, reverse MR analysis, and the ROC analysis for *ROMO1* and other typical biomarkers in diagnosing diseases. For detailed methods and results of our analyses, please refer to the “Supplementary methods and results material”.

## Discussion

The interplay between genetic factors and autoimmune diseases such as rheumatoid arthritis (RA), multiple sclerosis (MS), and type 2 diabetes (T1D) highlights the complexity of their shared pathogenetic. Emerging evidence indicates that these diseases not only share common genetic architectures but also exhibit significant comorbidity, suggesting underlying mechanisms that warrant further investigation. Understanding the genetic basis of these conditions can provide crucial insights into their etiology and progression (Rigante et al. 2014; Somers et al. 2009). Recent studies indicate that genetic variants associated with ROS may play crucial roles in the pathogenesis of these conditions (Hanaford and Johnson 2022; Miyata et al. 2020; Ghasemi et al. 2020). However, the precise mechanisms remain poorly understood. In this study, we employed a multi-omics approach, integrating multiple datasets including GEO, GWAS, scRNA-seq, and 256 ROSGs from MSigDB to identify and validate the shared genetic architecture in RA, MS, and T1D.

Initially, our study strongly suggested *ROMO1* as a key shared genetic structure of RA, MS, and T1D. Initially, through differential gene expression analysis combined with LASSO regression, we found that *ROMO1* was significantly differentially expressed in the whole blood transcriptome of the three autoimmune diseases. And multi-sample analysis further corroborated *ROMO1*’s significance by revealing its consistent association with immune cell patterns across tissues in all three diseases. Notably, significant associations between *ROMO1* and monocytes across different tissues in all three diseases. However, disease-specific differences were still observed. *ROMO1* exhibited varying associations with monocytes across the three autoimmune diseases (RA, Cor = 0.20, *P* = 0.037; MS, Cor = 0.41, *P* = 3.51 × 10^− 3^; T1D, Cor=-0.43, *P* = 3.93 × 10^− 3^).

Therefore, we supplemented our analysis with additional results. LDSC analysis revealed a negative correlation between RA and T1D (correlation coefficient = -0.93, *P* = 2.9 × 10^− 4^). Additionally, there was a slightly significant negative correlation between RA and MS (correlation coefficient = -0.46, *P* = 8.3 × 10^− 4^), and a slight but significant positive correlation between MS and T1D (correlation coefficient = 0.20, *P* = 0.041). These results explain that the three diseases themselves have inconsistent genetic associations. Meanwhile, MTAG analysis discovered that the shared risk locus *IL2RA* also showed distinct patterns among the three diseases. Importantly, *ROMO1* and *IL2RA* (Cor = -0.73, *P* < 0.001) exhibited significant correlations in autoimmune diseases. This finding is consistent with previous studies on the role of *IL2RA* in multiple autoimmune diseases, but our study is the first to reveal potential complex interactions between *ROMO1* and *IL2RA* (van Steenbergen et al. 2015; Buhelt et al. 2019; Qu et al. 2009).

In our evaluation of *ROMO1* as a diagnostic marker, we further revealed its complexity while highlighting its potential clinical applications. Through single-gene ROC analysis, we found that *ROMO1* performed well in the diagnosis of RA, demonstrating excellent diagnostic performance. In the diagnosis of MS, *ROMO1* showed moderate performance, but its diagnostic accuracy remained consistent. However, in the diagnosis of T1D, the performance of *ROMO1* was inconsistent. Our single-cell analysis corroborated these ROC results at the cellular level, providing an unprecedented high-resolution perspective on *ROMO1*’s role. This analysis not only confirmed the overall significant downregulation of *ROMO1* in RA and T1D patients but also precisely located this expression change primarily in CD4 + T cells, CD14 + HLA-DR + monocytes, and other immune cells. These findings at the cellular level emphasized the value of *ROMO1* as a potential diagnostic marker while revealing its potentially differentiated roles in various diseases. The high diagnostic accuracy of *ROMO1* in RA warrants further investigation.

Our study also provided strong evidence for *ROMO1*’s involvement in the ROS pathway in these three autoimmune diseases. Single-gene GSEA analysis revealed that *ROMO1* was significantly enriched in the KEGG ribosome pathway in all three diseases. GO analysis pointed to a negative correlation between *ROMO1* and the mitochondrial electron transport chain. These findings led us to speculate that *ROMO1* could be involved in the ROS pathway in RA, MS, and T1D. This insight was further substantiated by conjoint analysis, which identified *ROMO1* as the sole common ROSG across the diseases and demonstrated its similar immune patterns to known ROSGs. These results not only correspond to previous reports on the importance of oxidative stress and mitochondrial dysfunction in autoimmune diseases but also, for the first time, link *ROMO1* to the regulation of these key cellular physiological processes in an autoimmune context (Hanaford and Johnson 2022; Amini et al. 2019; Kwack et al. 2021).

Moreover, our study explored *ROMO1’*s potential mechanism in disease pathogenesis through monocyte function regulation. As previously mentioned, our immune infiltration analysis revealed significant associations between *ROMO1* and monocytes across different tissues in all three diseases. Analysis of six immune infiltration datasets showed consistent correlations between *ROMO1* and monocytes in whole blood and external samples. Importantly, *ROMO1* correlations maintained consistent signs within different tissue types of the same disease: for T1D, -0.43 in whole blood and − 0.42 in PBMCs; for RA, 0.20 in whole blood and 0.64 in PBMCs; and for MS, 0.41 in whole blood and 0.24 in white matter. This finding not only confirms previous research results but further elucidates the potential interaction between *ROMO1* and monocytes in autoimmune diseases (Sun et al. 2020; Wang et al. 2022; Rendra et al. 2019).

Further MR results underscored *ROMO1*’s role in regulating monocyte function across RA, MS, and T1D, while also reaffirming its complex nature. Upregulation of *ROMO1* expression was significantly associated with 14 monocyte-related indicators, showing a positive causal relationship with monocyte count and a negative causal relationship with HLA-DR + monocytes. Notably, HLA-DR + monocytes (especially CD14 + monocytes) had an OR greater than 1 for all three autoimmune diseases, suggesting their importance in increasing disease risk. Two-sample MR analysis further validated the causal relationships between *ROMO1* expression and these diseases, revealing a negative causal relationship with RA (suggesting a possible protective function) and a positive relationship with MS (indicating a risk factor). Finally in the supplemental results, we assessed the causal effect of monocytes on *ROMO1* expression by reverse MR analysis. The results showed a positive causal relationship between the number of monocytes and the upregulation of *ROMO1*, suggesting a bidirectional causal relationship between *ROMO1* and monocyte number. These findings not only emphasize the ability of *ROMO1* to regulate monocyte function in RA, MS, and T1D, but also reveal that there may be a complex mechanism of interaction between *ROMO1* and monocyte.

Finally, expanding on these findings, considering the key role of CD14 + HLA-DR + monocytes in antigen presentation and T cell activation, and our results suggesting that *ROMO1* may influence CD14 + HLA-DR + monocyte function in the pathogenesis of autoimmune diseases, we especially added supplementary results including integration analysis, cell communication analysis, and two-sample MR analysis to enrich and expand our understanding (Xue et al. 2021; Rigby et al. 1990; Casasola-LaMacchia et al. 2021). Our integration analysis revealed interconnections among immune cells across all diseases studied. Cell communication analysis showed that HLA-DR + monocytes primarily interacted with CD4 + T cells through the MIF signaling pathway, with four key genes (*CD44*, * CD74*, * MIF*, and *CXCR4*) expressed across multiple cell types in all three diseases. Two-sample MR analysis indicated a statistically significant association between increased MIF levels and a 17% higher risk of RA. These findings not only corroborate previous studies but also, for the first time, link *ROMO1* to the MIF signaling pathway, suggesting that *ROMO1* may modulate autoimmune responses by regulating CD14 + HLA-DR + monocyte function through this pathway, potentially affecting their ability to respond to pathogens and maintain immune homeostasis (Ma et al. 2019; Yukitake et al. 2017; Grieb et al. 2010).

Although our study provides important new insights, we must acknowledge that there are some limitations. First, this study serves as an exploratory study that initially identifies *ROMO1* as a potential target. While multi-organizational datasets provide a more comprehensive view of the study, additional variability is inevitably introduced. Meanwhile, this study mainly used bioinformatics analysis, and although the reproducibility of the correlations was strongly supported by statistical evidence and the false-positive rate was controlled for each method (Supplementary Table 17), computational multi-omics validation alone is still limited as conclusive evidence. Therefore, we suggest that follow-up studies should focus on specific tissue types for more in-depth mechanism exploration and functional validation to reduce systematic errors and accurately assess the direct impact of identified associations on disease mechanisms. Second, we used the regularization property of LASSO regression to screen for key genes in this study. However, despite our use of cross-validation to control the false-positive rate, LASSO regression still has limitations when dealing with highly correlated predictor variables and may miss key genes. Future studies could use methods more suitable for dealing with highly correlated predictor variables, such as elastic net regression, stability selection, or bayesian variable selection methods, to improve the reliability and interpretability of the study. Finally, regarding the MR methodology used in this study, although a causal link between *ROMO1* expression and disease was found using different methods, we must admit that the number of IVs for *ROMO1* is limited, which may affect the robustness of the results. Future studies with more IVs are needed to confirm these findings.

## Conclusion

In summary, our findings underscore the significance of *ROMO1* as a pivotal shared genetic element in RA, MS, and T1D. This study advances our understanding of the genetic interplay among these autoimmune diseases and opens up new avenues for research into their shared mechanisms. Future investigations should focus on elucidating the functional roles of *ROMO1* and its potential as a therapeutic target, thereby contributing to the development of personalized treatment strategies for affected individuals.

## Electronic supplementary material

Below is the link to the electronic supplementary material.

Supplementary Material 1

Supplementary Material 2

Supplementary Material 3

Supplementary Material 4

## Data Availability

The datasets provided in this study can be located in the following databases: GEO (http://www.ncbi.nlm.nih.gov/geo/), MSigDB (https://www.gsea-msigdb.org/), and GWAS (https://gwas.mrcieu.ac.uk). The source code for all the analyses in this study can be located in GitHub (https://github.com/TailinWang1/3-Autoimmune-Diseases).

## Abbreviations

RA: Rheumatoid arthritis
MS: Multiple sclerosis
GO: Gene ontology
HC: Healthy controls
MR: Mendelian randomization
T1D: Type 1 diabetes
GEO: Gene expression omnibus
DGE: Differential gene expression
AUC: Area under the curve
ROS: Reactive oxygen species
ROC: Receiver operating characteristic
ROMO1: Reactive Oxygen Species Modulator 1
eQTL: Expression quantitative trait locus
DEGs: Differentially expressed genes
KEGG: Kyoto encyclopedia of genes and genomes
GWAS: Genome-wide association studies
GSEA: Gene set enrichment analysis
ROSGs: ROS pathway-related genes
LASSO: Least absolute shrinkage and selection operator
MSigDB: Molecular Signatures Database
scRNA-seq: Single-cell RNA sequencing
