# Supplementary material for: A Multi-omics approach to identify and validate shared genetic architecture in rheumatoid arthritis, multiple sclerosis, and type 1 diabetes: integrating GWAS, GEO, MSigDB, and scRNA-seq data
Source: Funct Integr Genomics. 2025 Apr 21;25(1):91. doi: 10.1007/s10142-025-01598-x (PMC12009781; doi:10.1007/s10142-025-01598-x)
Supplement: Supplementary file 1 — Supplementary Material 1 [file 10142_2025_1598_MOESM1_ESM.docx]

**Supplementary Tables**

**Table S1**| Detailed information of included GEO data for single-gene ROC curve analysis.

| **Disease** | **GEO ID** | **Platform** | **Tissue** | **Case** | **Control** | **Total** |
| --- | --- | --- | --- | --- | --- | --- |
| RA | GSE205962 | GPL16043 | Whole blood | 16 | 4 | 20 |
| RA | GSE56649 | GPL570 | PBMCs | 13 | 9 | 22 |
| RA | GSE15573 | GPL6102 | PBMCs | 18 | 15 | 33 |
| MS | GSE21942 | GPL570 | PBMCs | 12 | 15 | 27 |
| MS | GSE108000 | GPL13497 | White matter | 30 | 10 | 40 |
| MS | GSE17048 | GPL6947 | Whole blood | 99 | 45 | 144 |
| T1D | GSE44314 | GPL6480 | Whole blood | 10 | 6 | 16 |
| T1D | GSE193273 | GPL20844 | PBMCs | 20 | 20 | 40 |
| T1D | GSE33440 | GPL6947 | PB monocyte | 16 | 6 | 22 |

**Table S2**| Detailed information of included GEO data for multi-sample analysis.

| **Disease** | **GEO ID** | **Platform** | **Tissue** | **Case** | **Control** | **Total** |
| --- | --- | --- | --- | --- | --- | --- |
| RA | GSE56649 | GPL570 | PBMCs | 13 | 9 | 22 |
| RA | GSE15573 | GPL6102 | PBMCs | 18 | 15 | 33 |
| MS | GSE21942 | GPL570 | PBMCs | 15 | 14 | 29 |
| MS | GSE108000 | GPL13497 | White matter | 30 | 10 | 40 |
| T1D | GSE193273 | GPL20844 | PBMCs | 20 | 20 | 40 |
| T1D | GSE110914 | GPL16791 | PB purified neutrophils | 26 | 16 | 42 |

**Table S3**| Detailed information of included 256 ROSGs data.

| **STANDARD_NAME** | GOBP_CELLULAR_RESPONSE_TO_OXIDATIVE_STRESS |
| --- | --- |
| **SYSTEMATIC_NAME** | M45123 |
| **COLLECTION** | C5:GO:BP |
| **MSIGDB_URL** | <https://www.gsea-msigdb.org/gsea/msigdb/human/geneset/GOBP_CELLULAR_RESPONSE_TO_OXIDATIVE_STRESS> |
| **NAMESPACE** | Human_NCBI_Gene_ID |
| **DESCRIPTION_BRIEF** | Any process that results in a change in state or activity of a cell (in terms of movement, secretion, enzyme production, gene expression, etc.) as a result of oxidative stress, a state often resulting from exposure to high levels of reactive oxygen species, e.g. superoxide anions, hydrogen peroxide (H^2^O^2^), and hydroxyl radicals. [GOC:mah] |
| **CONTRIBUTOR** | Gene Ontology |
| **CONTRIBUTOR_ORG** | Gene Ontology Consortium |
| **EXACT_SOURCE** | GO:0034599 |
| **EXTERNAL_DETAILS_URL** | <http://amigo.geneontology.org/amigo/term/GO:0034599> |
| **GENE_SYMBOLS** | *MIR675,HDAC6,IL18BP,PPIF,TRAP1,DHRS2,NET1,KLF2,RACK1,STK25,FBLN5,ARL6IP5,CAMKK2,PPARGC1A,PRDX3,RIPK3,ZNF277,PDCD10,TREX1,PARK7,CHUK,AGAP3,LRRK2,CPEB2,PPARGC1B,NCOA7,ATF2,SRXN1,ROMO1,SIRPA,PARP1,CRYGD,CYP1B1,VKORC1L1,DAPK1,DHFR,NQO1,ECT2,EDN1,EDNRA,EGFR,EIF2S1,AIF1,EPAS1,STX2,AKT1,ERN1,ETV5,EZH2,ABCD1,FABP1,FANCC,FANCD2,ALDH3B1,FER,PLA2R1,SIRT2,SETX,FOXO1,FOXO3,KDM6B,ATP13A2,SIRT1,FOS,SLC7A11,FXN,ALOX5,ABL1,FUT8,FYN,G6PD,MPV17L,PRDX5,SIN3A,ANKRD2,GCH1,GJB2,STAU2,FOXP1,HTRA2,H19,GPR37,GPX1,GPX5,GPX7,GSR,PYCR2,SLC25A24,ANXA1,HDAC2,HGF,HIF1A,HMOX1,NUDT2,HSF1,HSPA1A,HSPA1B,APOA4,IL6,AQP1,JUN,ERCC6L2,SUMO4,RHOB,TMIGD1,ARNT,BMAL1,MIRLET7B,MIR103A1,MIR107,MIR132,MIR133A1,MIR135A1,MIR17,MIR21,MIR34A,MIR92A1,MAPT,MDM2,MAP3K5,MET,MGAT3,MGST1,MMP2,MMP3,MMP9,MPO,MPV17,ABCC1,MSRA,MT3,MYB,NAGLU,ATF4,ATM,NFE2L2,NOS3,ATP2A2,DDR2,NR4A2,GPX8,PJVK,PRDX1,PNPLA8,PRKN,PAWR,PAX2,GLRX2,PCNA,CHCHD2,ZNF580,NME8,WNT16,RWDD1,OSER1,PDGFRA,PDK2,PENK,PEX10,PEX12,PEX13,PEX14,PKD2,ATP7A,RBM11,PPIA,TMEM161A,ADPRS,OXR1,ANKZF1,BRF2,SMPD3,PRKAA1,PRKAA2,AXL,PRKCD,SELENOS,PRKD1,MAPK1,MAPK3,MAPK7,MAPK8,MAPK9,MAPK13,SELENON,CBX8,DHFRP1,TBC1D24,MEAK7,PTPRK,PEX2,PXN,PEX5,PYCR1,RAD52,PLEKHA1,RELA,GAS5,RPS3,MAP2K4,PINK1,SLC1A1,NCF1,BMP7,SNCA,BNIP3,SOD1,SOD2,SOD3,SRC,STAT6,STAU1,STX4,BTK,PRDX2,TNFAIP3,TOP2B,TP53,TPM1,TRPM2,TXN,UCP1,VRK2,PCGF2,MAPKAP1,ZFAND1,PRR5L,PYROXD1,ERMP1,ZC3H12A,PDGFD,HM13,SESN2,SLC4A11,MAP1LC3A,CAT,MPV17L2,AIFM2,PRKRA,BECN1,PNPT1,RIPK1,IL18RAP,KAT2B,SPHK1,SQSTM1,TRPA1,SLC25A14,AIFM1,GPR37L1,LONP1,TP53INP1,CD36,KEAP1,CDK1,CCS* |
| **Version** | 2023.2.Hs: Updated to GO Release Oct 12, 2023. |

**Table S4**| Detailed information of included GEO data for conjoint analysis.

| **Disease** | **GEO ID** | **Platform** | **Tissue** | **Case** | **Control** | **Total** |
| --- | --- | --- | --- | --- | --- | --- |
| RA | GSE56649 | GPL570 | PBMCs | 13 | 9 | 22 |
| MS | GSE17048 | GPL6947 | Whole blood | 99 | 45 | 144 |
| T1D | GSE44314 | GPL6480 | Whole blood | 10 | 6 | 16 |

**Table S5|** Detailed information of scRNA-seq data.

| **Disease** | **GEO ID** | **Platform** | **Tissue** | **Case** |
| --- | --- | --- | --- | --- |
| RA | GSE159117 | GPL23227 | PBMCs | GSM4819747 |
| MS | GSE138266 | GPL18573 | PBMCs | GSM4104135 |
| T1D | GSE248284 | GPL16791 | PBMCs | GSM7910931 |
| HC | GSE244515 | GPL20795 | PBMCs | GSM7818500 |

**Table S6|** Detailed information of cell markers.

| **Cell** | **Marker** |
| --- | --- |
| B cells | CD79B,CD79A,CD74,PAX5,CD27(memory),IL4R(naive) |
| Monocytes | S100A8,S100A9,LYZ,CD14(CD14+),FCGR3A(CD16+),HLA-DRA(HLA-DR+),HLA-DRB1/5(HLA-DR+) |
| T cells | IL7R,CCR7,LTB,CD8A(CD8+),CD8B(CD8+),CD4(CD4+) |
| NK cells | GZMB,GZMH,NKG7 |
| Macrophages M2 | CD68,MERTK,AIF1,PSAP,CD163 |
| Mast cells | CPA3,GATA2 |
| Plasma cells | IGLL5,MZB1 |
| Megakaryocytes | GNG11,TUBB1 |

**Table S7|** Detailed information of GWAS data for two-sample MR analysis.

| **Phenotype** | **GWAS ID** | **Case** | **Control** | **Total** | **Ethnicity** |
| --- | --- | --- | --- | --- | --- |
| *ROMO1* | eqtl-a-ENSG00000125995 | NA | NA | 26,395 | European |
| RA | finn-b-RHEUMA_SEROPOS_OTH | 4,539 | 214,196 | 218,735 | European |
| MS | finn-b-G6_MS | 1,048 | 217,141 | 218,189 | European |
| T1D | ebi-a-GCST90014023 | 18,942 | 501,638 | 520,580 | European |
| Monocyte cell count | ebi-a-GCST90028998 | NA | NA | 545,193 | European |
| CCR2 on monocyte | ebi-a-GCST90002008 | NA | NA | 3,629 | European |
| CCR2 on CD14+ CD16- monocyte | ebi-a-GCST90002004 | NA | NA | 3,629 | European |
| CCR2 on CD14- CD16+ monocyte | ebi-a-GCST90001982 | NA | NA | 3,621 | European |
| CCR2 on CD14+ CD16+ monocyte | ebi-a-GCST90001992 | NA | NA | 3,618 | European |
| CD11c on monocyte | ebi-a-GCST90002089 | NA | NA | 2,805 | European |
| CD14 on CD14+ CD16- monocyte | ebi-a-GCST90001986 | NA | NA | 3,629 | European |
| CD14 on CD14+ CD16+ monocyte | ebi-a-GCST90001983 | NA | NA | 3,618 | European |
| CD16 on CD14+ CD16+ monocyte | ebi-a-GCST90002005 | NA | NA | 3,617 | European |
| CD16 on CD14- CD16+ monocyte | ebi-a-GCST90001979 | NA | NA | 3,620 | European |
| CD39 on monocyte | ebi-a-GCST90002034 | NA | NA | 2,909 | European |
| CD40 on monocyte | ebi-a-GCST90001985 | NA | NA | 3,629 | European |
| CD40 on CD14+ CD16- monocyte | ebi-a-GCST90001980 | NA | NA | 3,629 | European |
| CD40 on CD14- CD16+ monocyte | ebi-a-GCST90001989 | NA | NA | 3,621 | European |
| CD40 on CD14+ CD16+ monocyte | ebi-a-GCST90001981 | NA | NA | 3,618 | European |
| CD64 on monocyte | ebi-a-GCST90002006 | NA | NA | 3,622 | European |
| CD64 on CD14+ CD16- monocyte | ebi-a-GCST90001987 | NA | NA | 3,622 | European |
| CD64 on CD14- CD16+ monocyte | ebi-a-GCST90001990 | NA | NA | 3,614 | European |
| CD64 on CD14+ CD16+ monocyte | ebi-a-GCST90002011 | NA | NA | 3,611 | European |
| CD80 on monocyte | ebi-a-GCST90002039 | NA | NA | 2,850 | European |
| CD86 on monocyte | ebi-a-GCST90001905 | NA | NA | 2,850 | European |
| FSC-A on CD14+ monocyte | ebi-a-GCST90001967 | NA | NA | 3,112 | European |
| HLA-DR on monocyte | ebi-a-GCST90002010 | NA | NA | 3,629 | European |
| HLA-DR on CD14+ CD16- monocyte | ebi-a-GCST90001988 | NA | NA | 3,629 | European |
| HLA-DR on CD14+ CD16+ monocyte | ebi-a-GCST90002007 | NA | NA | 3,618 | European |
| PDL-1 on monocyte | ebi-a-GCST90002002 | NA | NA | 3,629 | European |
| PDL-1 on CD14+ CD16- monocyte | ebi-a-GCST90001993 | NA | NA | 3,629 | European |
| PDL-1 on CD14- CD16+ monocyte | ebi-a-GCST90001999 | NA | NA | 3,621 | European |
| PDL-1 on CD14+ CD16+ monocyte | ebi-a-GCST90001998 | NA | NA | 3,618 | European |
| SSC-A on CD14+ monocyte | ebi-a-GCST90002074 | NA | NA | 3,112 | European |

**Table S8|** Detailed information of external GWAS data for bidirectional MR analysis.

| **Disease** | **GWAS ID** | **Case** | **Control** | **Total** | **Ethnicity** |
| --- | --- | --- | --- | --- | --- |
| RA | ukb-a-105 | 3,730 | 333,429 | 337,159 | European |
| MS | finn-b-G6_MS | 1,048 | 217,141 | 218,189 | European |
| T1D | ebi-a-GCST90014023 | 18,942 | 501,638 | 520,580 | European |

**Table S9|** Detailed information of GWAS data for LDSC analysis.

| **Disease** | **GWAS ID** | **Case** | **Control** | **Total** | **Ethnicity** |
| --- | --- | --- | --- | --- | --- |
| RA | GCST90132226 | 17,221 | 74,823 | 92,044 | European |
| RA | GCST90132223 | 22,350 | 74,823 | 97,173 | European |
| MS | GCST003566 | 4,888 | 10,395 | 15,283 | European |
| MS | GCST001198 | 9,772 | 16,849 | 26,621 | European |
| MS | ieu-a-1025 | 14,498 | 24,091 | 38,589 | European |
| T1D | GCST005536 | 6,683 | 12,173 | 29,652 | European |

**Table S10|** Detailed information of included GEO data for correlation analysis

| **Disease** | **GEO ID** | **Platform** | **Tissue** | **Case** | **Control** | **Total** |
| --- | --- | --- | --- | --- | --- | --- |
| RA | GSE205962 | GPL16043 | Whole blood | 16 | 4 | 20 |
| MS | GSE23832 | GPL6244 | PBMCs | 8 | 4 | 12 |
| T1D | GSE193273 | GPL20844 | PBMCs | 20 | 20 | 40 |

**Table S11|** Detailed information of external GWAS data for two-sample MR analysis

| **Phenotype** | **GWAS ID** | **Case** | **Control** | **Total** | **Ethnicity** |
| --- | --- | --- | --- | --- | --- |
| Macrophage migration inhibitory factor(MIF) | prot-c-5356_2_3 | NA | NA | NA | European |
| RA | finn-b-RHEUMA_SEROPOS_OTH | 4,539 | 214,196 | 218,735 | European |
| MS | finn-b-G6_MS | 1,048 | 217,141 | 218,189 | European |
| T1D | ebi-a-GCST90014023 | 18,942 | 501,638 | 520,580 | European |

**Table S12|** Detailed information of typical biomarkers in RA, MS and T1D.

| **Disease** | **Biomarker** | **Reference** |
| --- | --- | --- |
| RA | *CRP* | <https://pubmed.ncbi.nlm.nih.gov/35275765/> |
|  | *ADA* | <https://pmc.ncbi.nlm.nih.gov/articles/PMC7765045/> |
| MS | *CXCL13* | <https://pubmed.ncbi.nlm.nih.gov/23322500/> |
|  | *CXCL5* | <https://pmc.ncbi.nlm.nih.gov/articles/PMC10331241/> |
| T1D | *TYK2* | <https://pmc.ncbi.nlm.nih.gov/articles/PMC9606380/> |
|  | *TCL1A* | <https://pmc.ncbi.nlm.nih.gov/articles/PMC11872515/> |

**Table S13|** The causal associations between RA,MS and T1D.

| **Exposure** | **Methods** | **Snps** | **OR** | **Low** | **High** | **P** | **Het** | **Ple** | **Outcome** |
| --- | --- | --- | --- | --- | --- | --- | --- | --- | --- |
| RA | MR Egger | 8 | 2.15E+68 | 6.03E+29 | 7.69E+106 | 0.013 | 1.10E-203 | 0.73 | T1D |
| RA | Weighted median | 8 | 1.58E+18 | 5.78E+11 | 4.32E+24 | 7.79E-09 |  |  | T1D |
| RA | IVW | 8 | 2.74E+62 | 3.89E+41 | 1.94E+83 | 4.36E-09 | 3.25E-207 |  | T1D |
| RA | Simple mode | 8 | 3.57E+06 | 5.91E+00 | 2.16E+12 | 0.065 |  |  | T1D |
| RA | Weighted mode | 8 | 2.09E+10 | 2.35E+04 | 1.86E+16 | 0.0098 |  |  | T1D |
| MS | MR Egger | 5 | 0.16 | 0.02 | 1.22 | 0.17 | 2.07E-135 | 0.32 | T1D |
| MS | Weighted median | 5 | 0.81 | 0.74 | 0.88 | 3.37E-06 |  |  | T1D |
| MS | IVW | 5 | 0.51 | 0.26 | 0.99 | 0.047 | 3.90E-198 |  | T1D |
| MS | Simple mode | 5 | 0.84 | 0.75 | 0.93 | 0.041 |  |  | T1D |
| MS | Weighted mode | 5 | 0.82 | 0.75 | 0.89 | 0.014 |  |  | T1D |
| T1D | MR Egger | 85 | 0.86 | 0.78 | 0.94 | 0.0027 | 2.20E-35 | 0.13 | MS |
| T1D | Weighted median | 85 | 0.90 | 0.86 | 0.96 | 5.20E-04 |  |  | MS |
| T1D | IVW | 85 | 0.90 | 0.83 | 0.97 | 6.55E-03 | 9.62E-37 |  | MS |
| T1D | Simple mode | 85 | 0.90 | 0.72 | 1.12 | 0.31 |  |  | MS |
| T1D | Weighted mode | 85 | 0.90 | 0.85 | 0.95 | 4.59E-04 |  |  | MS |
| RA | MR Egger | 7 | 5.13E-14 | 3.50E-36 | 7.52E+08 | 0.29 | 4.48E-04 | 0.59 | MS |
| RA | Weighted median | 7 | 4.23E-10 | 9.04E-17 | 1.98E-03 | 0.0046 |  |  | MS |
| RA | IVW | 7 | 1.35E-08 | 3.37E-20 | 5.44E+03 | 0.18 | 5.59E-04 |  | MS |
| RA | Simple mode | 7 | 3.23E+04 | 1.05E-14 | 9.88E+22 | 0.66 |  |  | MS |
| RA | Weighted mode | 7 | 4.50E-10 | 8.53E-16 | 2.37E-04 | 0.021 |  |  | MS |
| MS | MR Egger | 4 | 0.99 | 0.99 | 1.00 | 0.35 | 0.0076 | 0.70 | RA |
| MS | Weighted median | 4 | 0.99 | 0.99 | 0.99 | 5.88E-14 |  |  | RA |
| MS | IVW | 4 | 0.99 | 0.99 | 0.99 | 9.23E-07 | 0.013 |  | RA |
| MS | Simple mode | 4 | 0.99 | 0.99 | 0.99 | 0.037 |  |  | RA |
| MS | Weighted mode | 4 | 0.99 | 0.99 | 0.99 | 4.80E-03 |  |  | RA |
| T1D | MR Egger | 85 | 1.002 | 1.002 | 1.003 | 7.25E-12 | 6.47E-14 | 0.051 | RA |
| T1D | Weighted median | 85 | 1.002 | 1.002 | 1.003 | 4.22E-22 |  |  | RA |
| T1D | IVW | 85 | 1.002 | 1.002 | 1.002 | 6.88E-20 | 4.05E-15 |  | RA |
| T1D | Simple mode | 85 | 0.99 | 0.99 | 1.00 | 0.96 |  |  | RA |
| T1D | Weighted mode | 85 | 1.002 | 1.001 | 1.003 | 1.66E-08 |  |  | RA |

**Table S14|** The genetic correlations between RA,MS and T1D.

| **Trait1** | **Trait2** | **r_g_** | **SE** | **P-value** |
| --- | --- | --- | --- | --- |
| RA | T1D | -0.90 | 0.25 | 3.6×10^-4^ |
|  |  | -0.93 | 0.26 | 2.9×10^-4^ |
| MS | T1D | 0.67 | 0.42 | 0.11 |
|  |  | 0.82 | 0.60 | 0.17 |
|  |  | 0.20 | 0.093 | 0.041 |
| MS | RA | -0.22 | 0.078 | 4.8×10^-3^ |
|  |  | -0.46 | 0.14 | 8.3×10^-4^ |
|  |  | -0.37 | 0.20 | 0.064 |
|  |  | -0.17 | 0.072 | 0.016 |
|  |  | -0.40 | 0.13 | 2.1×10^-3^ |
|  |  | -0.37 | 0.19 | 0.045 |

**Table S15|** The effect estimates for the causal influence of MIF on the risk of RA.

| **Exposure** | **Method** | **N** | **OR(95%CI)** | **P values** | **Het** | **Ple** | **Outcome** |
| --- | --- | --- | --- | --- | --- | --- | --- |
| MIF | Weighted median | 3 | 1.17(1.02,1.34) | 0.02 | 0.88 | 0.73 | RA |
|  | IVW | 3 | 1.17(1.04,1.31) | 0.01 |  |  |  |
|  | Simple mode | 3 | 1.18(1.01,1.39) | 0.16 |  |  |  |
|  | Weighted mode | 3 | 1.17(1.00,1.37) | 0.19 |  |  |  |

**Table S16|** The effect estimates for the causal influence of monocyte cell count on the risk of *ROMO1.*

| **Exposure** | **Method** | **N** | **OR(95%CI)** | **P values** | **Het** | **Ple** | **Outcome** |
| --- | --- | --- | --- | --- | --- | --- | --- |
| Monocyte cell count | MR Egger | 157 | 1.13(1.02,1.26) | 0.019 | 0.85 | 0.83 | *ROMO1* |
|  | Weighted median | 157 | 1.13(1.02,1.26) | 0.020 |  |  |  |
|  | IVW | 157 | 1.14(1.07,1.22) | 4.47E-05 |  |  |  |
|  | Simple mode | 157 | 1.07(0.85,1.35) | 0.55 |  |  |  |
|  | Weighted mode | 157 | 1.18(1.03,1.34) | 0.014 |  |  |  |

**Table S17|** The approaches to controlling type I errors (false positive rate) throughout the workflow process.

| **Analysis** | **Approaches to controlling false-positive rate** |
| --- | --- |
| DGE analysis | False positive detection rate (FDR) correction. |
| LASSO regression | Cross-validation was performed to select the value of λ that minimizes the model error, and ROC values were calculated to jointly reduce the risk of false positives. |
| Single-gene GSEA | A 10,000 permutations test was used to assess the significance of the gene set, while the minimum and maximum size of the gene set was set (10 and 200, respectively) to exclude gene sets that were too small or too large, and finally the GSEA algorithm provided adjusted P-values to control for false positives from multiple hypothesis testing. |
| Single-gene ROC analysis | The false-positive rate was effectively controlled through the steps of evaluating AUC, independent dataset validation and visual analysis. |
| Immune infiltration analysis | The false-positive rate was controlled by randomly disrupting the sample labels and recalculating the proportion of immune cells to assess whether the observed differences were statistically significant. |
| Single-cell analysis | Low-quality cells were removed by setting strict threshold screening conditions (e.g., 200 < nFeature_RNA < 2500 and percent.mt < 5%) at the initial stage of data processing, and a further two-cell removal step was implemented for datasets containing more than 10,000 cells. |
| MR analysis | SNPs with a P-value of less than 5×10^-^⁶were selected as instrumental variables, F-statistics were calculated, weak instrumental variables were excluded, the chain disequilibrium parameter r²<0.001 was set and a physical distance window of 100 kb was used, and finally multiple sensitivity analyses were performed to collectively control for false-positive rates. |
