# Supplementary material for: A Multi-omics approach to identify and validate shared genetic architecture in rheumatoid arthritis, multiple sclerosis, and type 1 diabetes: integrating GWAS, GEO, MSigDB, and scRNA-seq data
Source: Funct Integr Genomics. 2025 Apr 21;25(1):91. doi: 10.1007/s10142-025-01598-x (PMC12009781; doi:10.1007/s10142-025-01598-x)
Supplement: Supplementary file 2 — Supplementary Material 2 [file 10142_2025_1598_MOESM2_ESM.docx]

**Supplementary Methods and Results**

**Methods**

**Identification of causal associations in RA, MS, and T1D through bidirectional MR analysis**

To identify the existence of causal associations between autoimmune diseases, we focused on the bidirectional MR analysis among the 3 autoimmune diseases using the "TwoSampleMR" R package (**Supplementary Table 8**) (Xu et al. 2023; Nica & Dermitzakis 2013). IVs were selected using a threshold of standards: P < 5×10^−8^, kb = 10,000, r^2^ < 0.001, and F-statistic >20. All results were subjected to heterogeneity (het) and pleiotropy (ple) tests to ensure the robustness and reliability of our causal inferences (Hemani et al. 2018). And the significance threshold was P-value < 0.05.

**Identification of genetic correlations in RA, MS, and T1D through LDSC analysis**

We conducted a LDSC analysis using the GWAS datasets for the three diseases to identify the genetic correlations among these diseases (**Supplementary Table 9**). Specifically, we used the “MRlap” R package for the analysis (Fang et al. 2023). This package offers robust tools for genetic correlation estimation and is particularly well-suited for analyzing complex trait relationships. Our analysis included multiple GWAS datasets for each disease to ensure comprehensive coverage and enhance the reliability of our findings.

**Identification of shared genetic variants in RA, MS, and T1D through Multi-Trait Analysis of GWAS (MTAG)**

After the LDSC analysis, we further utilized MTAG (Multi-Trait Analysis of GWAS) to integrate GWAS results from these three diseases (Xu et al. 2023). Data sources included GWAS summary statistics from **Supplementary Table 9**: MS (GCST003566), RA (GCST90132226), and T1D (GCST005536). MTAG analysis was conducted in a Python environment using packages such as “scipy”, “pandas”, “argparse”, “bitarray”, and “joblib” (Villavicencio et al. 2022). The results were then visualized in R using the “qqman” package (Adam et al. 2021). Finally, we applied a p-value threshold of 5×10^−5^ to identify significant SNPs and recognized shared risk loci among the three diseases.

**Identification of relationship between *ROMO1 a*nd *IL2RA* in RA, MS, and T1D through correlation analysis**

Our previous research identified *ROMO1* as a shared genetic factor among RA, MS, and T1D. However, through immune infiltration and MR analyses, we discovered that *ROMO1* exhibits different behaviors in these three diseases. Further correlation analysis revealed that the SNP rs12722563, associated with the *IL2RA* gene, shows a protective effect in RA but acts as a risk factor in MS and T1D. Based on these findings, we hypothesized a potential relationship between *IL2RA* and *ROMO1*. To test this hypothesis, we conducted a correlation analysis of *ROMO1* and *IL2RA* for the three diseases **(Supplementary Table 10**). We used the 'rcorr' function from the 'Hmisc' R package to calculate Pearson correlation coefficients and their corresponding P-values (Baum et al. 2019). And the significance threshold was P-value < 0.05. Then we used the “ggplot2” package to generate heatmaps that visually represent the strength and significance of correlations between genes (Ito & Murphy 2013).

**Identification of cell-cell relationship in RA, MS, and T1D through integration analysis**

We integrated scRNA-seq data from the three autoimmune diseases and performed integrated cell clustering (**Supplementary Table 5)**. We employed Uniform Manifold Approximation and Projection (UMAP) for dimensionality reduction and visualization (Becht et al. 2018). Then we updated metadata, calculated average gene expression levels for each cell type, and selected the top 2000 genes with the highest expression variance (Itai et al. 2023). Using these genes, we computed Spearman correlation coefficients between cell types and generated a heatmap using the "pheatmap" R package to visualize relationships between different cell populations (Li et al. 2022).

**Identification of cell-cell communication in RA, MS, and T1D through cell communication analysis**

Using the "CellChat" R package, we analyzed single-cell RNA sequencing data from the three diseases, focusing on interaction patterns between CD14+HLA-DR+ and CD16+HLA-DR+ monocytes and other cell types (**Supplementary Table 5)** (Jin et al. 2021). We constructed cell-cell communication networks across RA, MS, and T1D, and performed enrichment analysis of communication pathways. We particularly examined the communication networks involving CD14+ HLA-DR+ monocytes, CD16+ HLA+ monocytes, and CD4+ T cells. We conducted an in-depth analysis of the MIF pathway communication, generated violin plots showing the expression of MIF pathway components (*CD44, CD74, CXCR4, MIF*) across all cell types, and illustrated sender-receiver relationships in the MIF pathway across different cell types.

**Identification of causal relationships between MIF and three autoimmune diseases through two-sample MR analysis**

To assess the causal relationships between MIF and three autoimmune diseases, we conducted a two-sample MR analysis using the 'TwoSampleMR' R package (Xu et al. 2023). We utilized GWAS data for MIF (prot-c-5356_2_3) and for the three autoimmune diseases for our analysis (**Supplementary Table 11**). In selecting IVs, we applied stringent criteria: a significance level of P-values < 5×10^−6^, a linkage disequilibrium window size of 10,000 base pairs, a linkage disequilibrium coefficient r² < 0.001, and an F-statistic greater than 20. After screening, we identified 3 SNPs that met these conditions as our instrumental variables.To ensure the robustness and reliability of our causal inferences, all results underwent heterogeneity and pleiotropy tests.

**Identification of causal relationships between *ROMO1* and monocytes through reverse MR analysis**

In previous studies, forward MR analysis had shown that *ROMO1*, as an exposure factor, had a significant causal effect on certain monocytes and their phenotypes. To more fully understand the mechanism of interaction between *ROMO1* and monocytes, we further performed the reverse MR analysis with monocytes as the exposure factor and *ROMO1* as the outcome variable to assess the causal effect of monocytes on *ROMO1* expression (**Supplementary Table 7**). In the reverse MR analysis, we applied stringent IV screening criteria, including P value < 5×10^-8^, kb = 10,000, and r^2^ < 0.001. All results were tested for heterogeneity (het) and multiplicity (ple) to ensure the robustness and reliability of causal inference. The significance threshold was set at a P value < 0.05.

**Identification of differences in diagnostic performance between *ROMO1* and common markers of autoimmune disease through ROC curve analysis**

In the initial analysis, we performed single-gene ROC curve analysis from the GEO dataset as a way to evaluate the validity of key shared genes as potential diagnostic markers for RA, MS and T1D. For each disease, we set up one training set and two validation sets as follows: RA (training set: GSE56649, validation sets: GSE15573, GSE205962), MS (training set: GSE21942, validation sets: GSE108000, GSE17048), and T1D (training set: GSE44314. validation set: GSE19273, GSE33440) (**Supplementary Table 1**). To further enhance the credibility of the findings, we reviewed the relevant literature in recent years, screened the common biomarkers of RA, MS and T1D (**Supplementary Table 12**), and performed ROC curve analysis on them to assess the diagnostic performance and compare them with the diagnostic efficacy of *ROMO1*. In this process, we followed the analytical and quality control methods of the single-gene ROC curve analysis part of the main analysis.

**Results**

**Identification of causal associations in RA, MS, and T1D through bidirectional MR analysis**

The MR analysis (**Supplementary Table 13 and Supplementary Figure 3**) revealed the causal associations between RA, MS, and T1D, confirming the presence of causality among these autoimmune diseases. This finding provides supporting evidence for the previously observed comorbidity among these three autoimmune disorders, offering a genetic basis for their co-occurrence (Lernmark 2002; Hojjati et al. 2016; Liao et al. 2009).

**Identification of genetic correlations in RA, MS, and T1D through LDSC analysis**

Building upon these causal relationships, the LDSC regression analysis further revealed significant genetic correlations among the three diseases (**Supplementary Table 14 and Supplementary Figure 4A**). There was a negative correlation between RA and T1D(correlation coefficient = -0.93, P = 2.9×10^-4^). Additionally, there was a slightly significant negative correlation between RA and MS(correlation coefficient = -0.46, P = 8.3×10^-4^), and a slight but significant positive correlation between MS and T1D (correlation coefficient = 0.20, P = 0.041). These results not only corroborate the shared genetic basis between the three diseases as indicated by the MR analysis, but also provide a more nuanced understanding of their genetic relationships.

**Identification of shared genetic variants in RA, MS, and T1D through Multi-Trait Analysis of GWAS (MTAG)**

The Manhattan plots from MTAG analysis illustrated genetic associations for RA, MS, and T1D(**Supplementary Figure 4B,C,D)**. Using a P-value threshold of 5×10^-5^, we identified one SNP, rs12722563, significantly associated with all three diseases. This SNP is located on chromosome 10 at position 6069561. This SNP is associated with the *IL2RA* gene, which has been widely shown to be associated with a variety of autoimmune diseases (Frederiksen et al. 2013). This finding further supported the idea that there was a common genetic factor. Notably, SNP rs12722563 exhibited a protective effect in RA (effect size -0.022, P =1.67×10^-5^), while showing risk effects in MS (effect size 0.101, P = 3.92×10^-8^) and T1D (effect size 0.117, P =3.04×10^-20^).

**Identification of relationship between *ROMO1 a*nd *IL2RA* in RA, MS, and T1D through correlation analysis**

The correlation analysis results confirmed our hypothesis: *ROMO1* showed a significant negative correlation with *IL2RA* in RA (Cor = -0.73, P < 0.001), while exhibiting positive correlations in T1D and MS (Cor = 0.22 and 0.46) (**Supplementary Figure 4E,F,G)**. These results provided additional explanation for *ROMO1*'s role as an important shared genetic factor among the three diseases and its varying manifestations across different conditions.

**Identification of cell-cell relationship in RA, MS, and T1D through integration analysis**

By integrating single-cell data from the three diseases, we successfully identified 11 distinct cell clusters, and the correlation heatmap provided insights into the relationships among these 11 immune cell populations (**Supplementary Figure 5A,B**).

**Identification of cell-cell communication in RA, MS, and T1D through cell communication analysis**

The results indicated that monocytes primarily communicated with CD4+ T cells, and this communication was mainly mediated through the macrophage migration inhibitory factor (MIF) signaling pathway(**Supplementary Figure 5C-I**). Further analysis revealed that four key genes in the MIF pathway (*CD44, CD74, MIF,* and *CXCR4*) were expressed in multiple cell types across these three diseases(**Supplementary Figure 5J**). In the MIF pathway, CD4+ T cells were the main signal senders, while CD14+HLA-DR+ monocytes were the primary signal receivers(**Supplementary Figure 5K**). Overall, these findings provided a more in-depth molecular-level explanation of its mechanism of action.

**Identification of causal relationships between MIF and three autoimmune diseases through two-sample MR analysis**

The analysis revealed that in the MR analysis between MIF and RA, the OR was 1.17 (95% CI: 1.04, 1.31) with a P-value of 0.01 (**Supplementary Table 15 and Supplementary Figure 6)**. This result indicated that elevated levels of MIF were associated with a 17% increased risk of RA, and this association was statistically significant. This finding provided strong evidence for the important role of MIF in the development of autoimmune diseases, particularly RA.

**Identification of causal relationships between *ROMO1* and monocytes through reverse MR analysis**

In the reverse MR analysis, the results showed a significant positive causal relationship between monocyte counts and *ROMO1* (**Supplementary Table 16 and Supplementary Figure 7**). This finding suggested that there may be a complex mechanism of interaction between *ROMO1* and monocyte number; *ROMO1* may promote monocyte production or survival through some pathway, whereas an increase in monocyte number may further up-regulate *ROMO1* expression through a feedback mechanism.

**Identification of differences in diagnostic performance between *ROMO1* and common markers of autoimmune disease through ROC curve analysis**

In the diagnostic performance evaluation of RA (**Supplementary Figure 8**), *ROMO1* reached an AUC of 0.991 in the training set, indicating its extremely high diagnostic accuracy in the training phase. In validation set 1, the AUC of *ROMO1* was 0.781, which decreased but reached 1.000 in validation set 2, showing its stability and reliability on different datasets. In contrast, the training set AUC of *CRP* was only 0.556, 0.507 in validation set 1, and 0.891 in validation set 2, although there was a high value of 0.891, but the overall fluctuation was large and the stability was insufficient. The performance of *ADA* was even worse, with an AUC of 0.581 for the training set, 0.526 in validation set 1, and 0.984 in validation set 2, although there were also significant fluctuations, the unstable diagnostic performance.

In the diagnostic performance evaluation of MS (**Supplementary Figure 9**), the training set AUC of *ROMO1* reached 0.910, indicating its high diagnostic accuracy in the training phase. In validation set 1 and validation set 2, the AUCs of *ROMO1* were 0.723 and 0.720, respectively, which decreased from the training set but maintained a relatively stable and consistent performance in the two independent validation sets, indicating its good generalization ability and reliability. In contrast, the training set AUC of *CXCL13* was 0.738, which was lower than the training set performance of *ROMO1*. Although *CXCL13* achieved a high AUC value of 0.903 in validation set 1, the AUC in validation set 2 plummeted to 0.490, which was extremely unstable. The overall performance of *CXCL5* was even worse, with an AUC of 0.583 in the training set, and AUC values of 0.653 and 0.585 in the two validation sets, which were lower than that of *ROMO1*.

In the diagnostic performance evaluation of T1D (**Supplementary Figure 10**), *ROMO1* reached an AUC of 1.000 in the training set, indicating its perfect diagnostic accuracy in the training phase. In validation set 1, the AUC of *ROMO1* was 0.594, which decreased but rebounded to 0.708 in validation set 2, showing some stability. In contrast, *TYK2* performed better with an AUC of 0.950 in the training set, but dropped to 0.623 in validation set 1 and 0.698 in validation set 2, which was more fluctuating and less stable than that of *ROMO1*. The overall performance of *TCL1A*, on the other hand, lagged significantly behind, with an AUC of 0.567 in the training set, and AUCs of 0.448 in validation set 1 and 0.625 in validation set 2, both of which were lower than that of *ROMO1*, indicating its poor diagnostic performance.

In conclusion, *ROMO1* demonstrated superior diagnostic performance compared to other biomarkers for the three autoimmune diseases, RA, MS, and T1D. However, although we observed perfect classification (AUC equal to 1) in RA and T1D, we had to consider that it was a matter of insufficient sample size, so these results should be considered with caution.
