# Supplementary material for: A Multi-omics approach to identify and validate shared genetic architecture in rheumatoid arthritis, multiple sclerosis, and type 1 diabetes: integrating GWAS, GEO, MSigDB, and scRNA-seq data
Source: Funct Integr Genomics. 2025 Apr 21;25(1):91. doi: 10.1007/s10142-025-01598-x (PMC12009781; doi:10.1007/s10142-025-01598-x)
Supplement: Supplementary file 3 — Supplementary Material 3 [file 10142_2025_1598_MOESM3_ESM.docx]

**Supplementary Figures**

**

**

**Figure S1**

The Venn diagram displays the 8 shared gene(*ROMO1, FAU, RPS10, RPL35, RPS17, RPS27, LSM3, and RPL27)* among RA, MS and T1D.


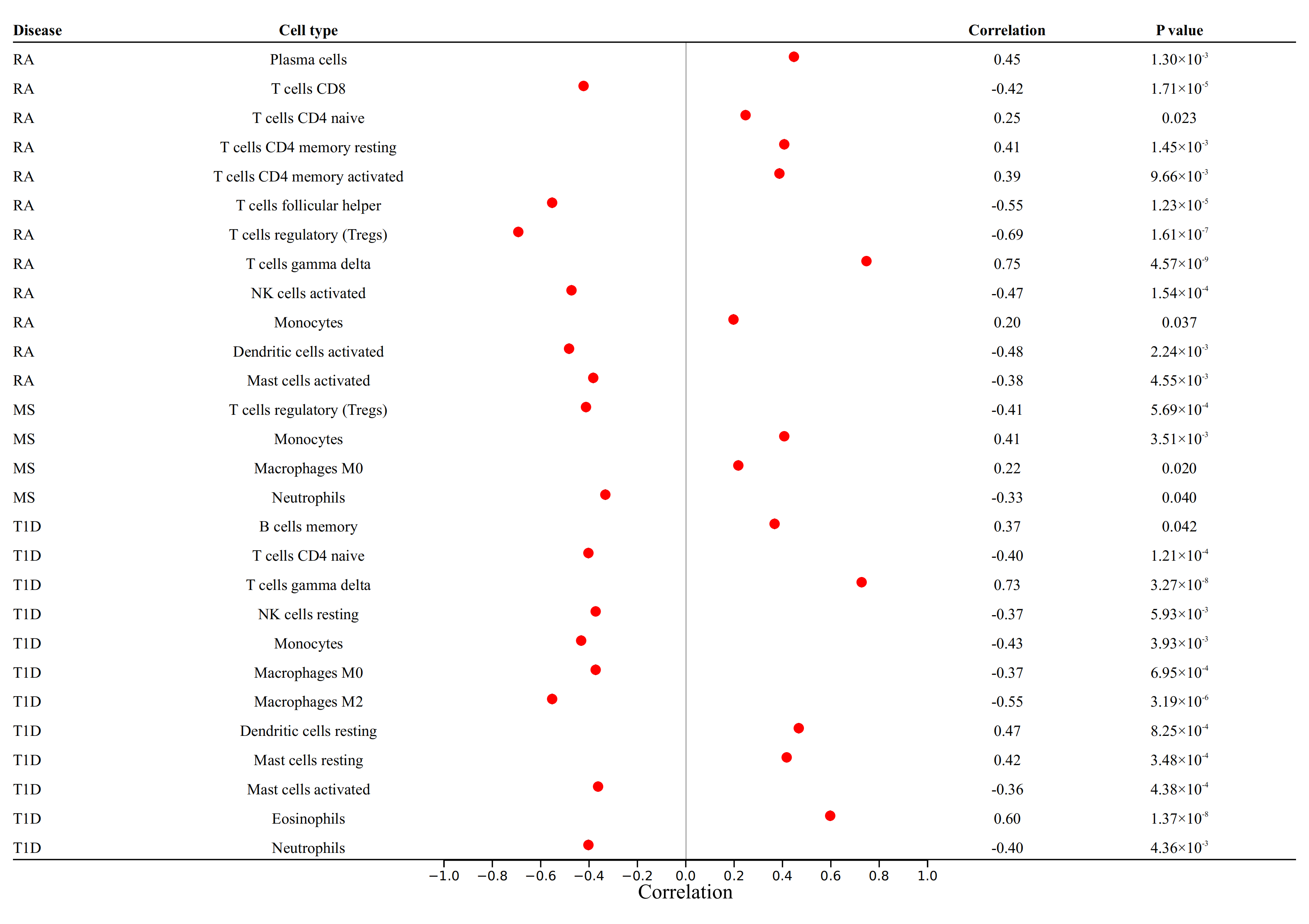


**Figure S2**

The significant correlations between *ROMO1* and RA, MS and T1D immune cell subsets.

**
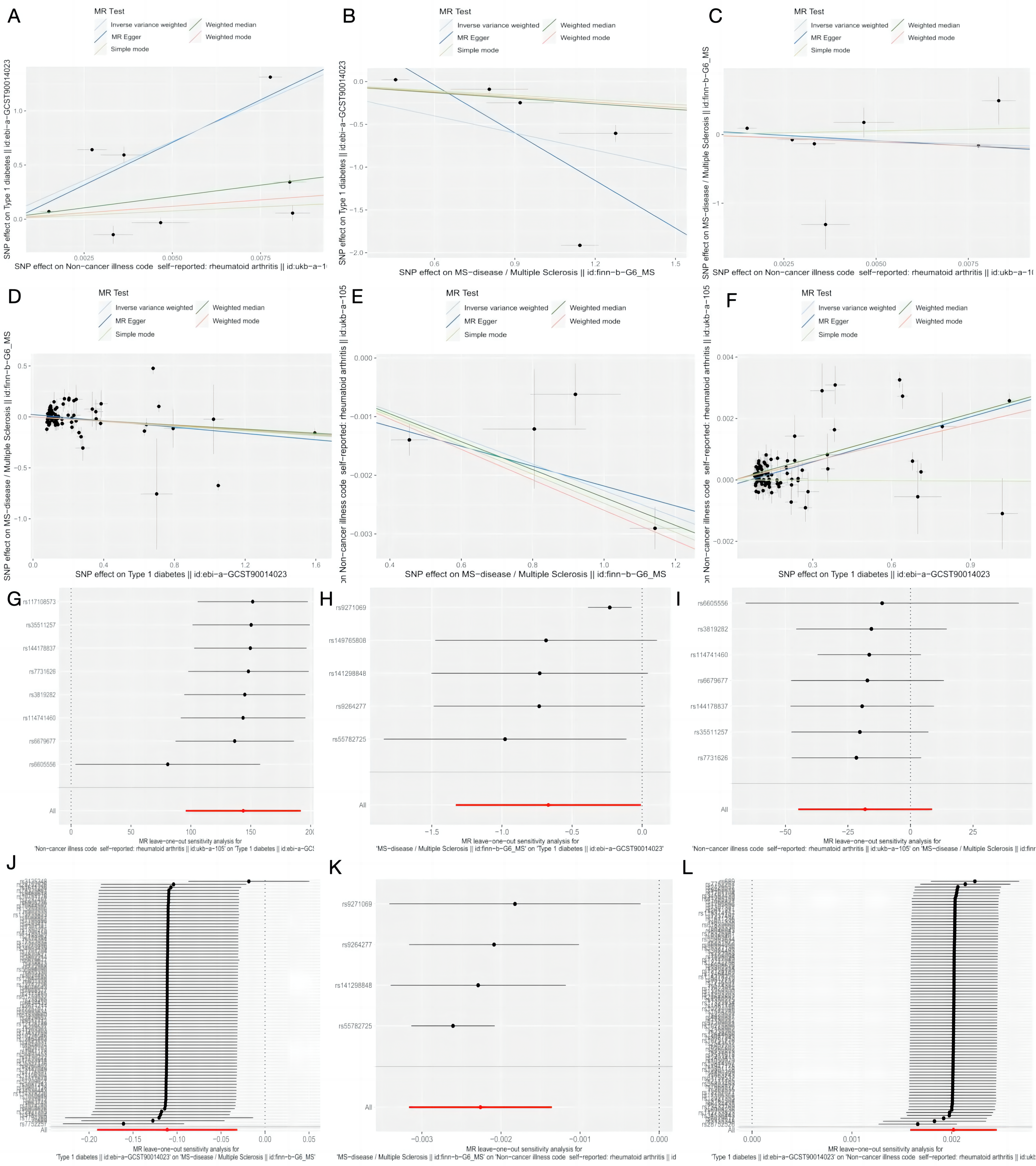
Figure S3**

(A) The scatter plot displays the effect estimates for the causal effect of RA on the risk of T1D.(B) The scatter plot displays the effect estimates for the causal effect of MS on the risk of T1D. (C) The scatter plot displays the effect estimates for the causal influence of RA on the risk of MS. (D) The scatter plot displays the effect estimates for the causal effect of T1D on the risk of MS. (E) The scatter plot displays the effect estimates for the causal influence of MS on RA risk. (F) The scatter plot displays the effect estimates for the causal effect of T1D on RA risk. (G) Leave-one-out analysis plots for RA on T1D.(H) Leave-one-out analysis plots for MS on T1D. (I) Leave-one-out analysis plots for RA on MS. (J) Leave-one-out analysis plots for T1D on MS. (K) Leave-one-out analysis plots for MS on RA. (L) Leave-one-out analysis plots for T1D on RA.





**Figure S4**

(A) The genetic correlations among RA, MS and T1D. (B) The Manhattan plot for RA. (C) The Manhattan plot for MS. (D) The Manhattan plot for T1D. (E) The heatmap showing the relationship between *IL2RA* and *ROMO1* in RA(*P < 0.05, **P < 0.01, ***P < 0.001). (F) The heatmap showing the relationship between *IL2RA* and *ROMO1* in MS(*P < 0.05, **P < 0.01, ***P < 0.001). (G) The heatmap showing the relationship between *IL2RA* and *ROMO1* in T1D(*P < 0.05, **P < 0.01, ***P < 0.001).

**

**

**Figure S5**

(A)The integrated analysis of RA/MS/T1D. (B) The correlation analysis of cell clusters across RA, MS, and T1D. (C,D) The networks of cell-cell communication cross RA, MS, and T1D. (E)The enrichment analysis of cell communication pathways. (F-H) The cell-cell communication network for CD14+ HLA-DR+ monocytes, CD16+ HLA+ monocytes, and CD4+ T cells. (I) The cell-cell communication analysis focusing on CD14+ HLA-DR+ monocytes, CD16+ HLA+ monocytes, and CD4+ T cells. (J) The violin plots showing the expression of MIF pathway components (*CD44, CD74, CXCR4, MIF*) across all cell types. (K) The sender-receiver relationships in the MIF pathway across different cell types.

**
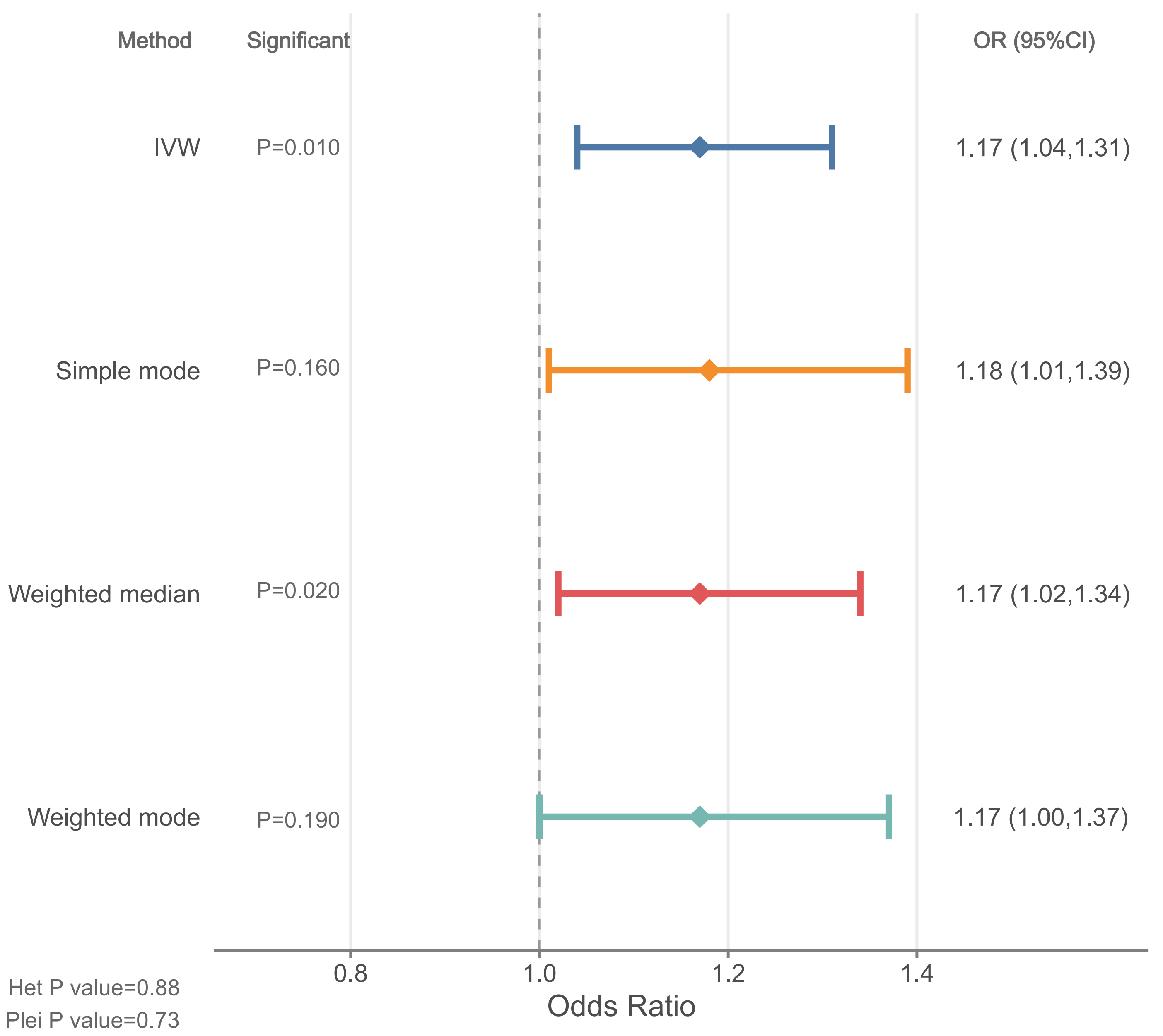
**

**Figure S6**

The forest plot displays the effect estimates for the causal influence of MIF on the risk of RA.

**
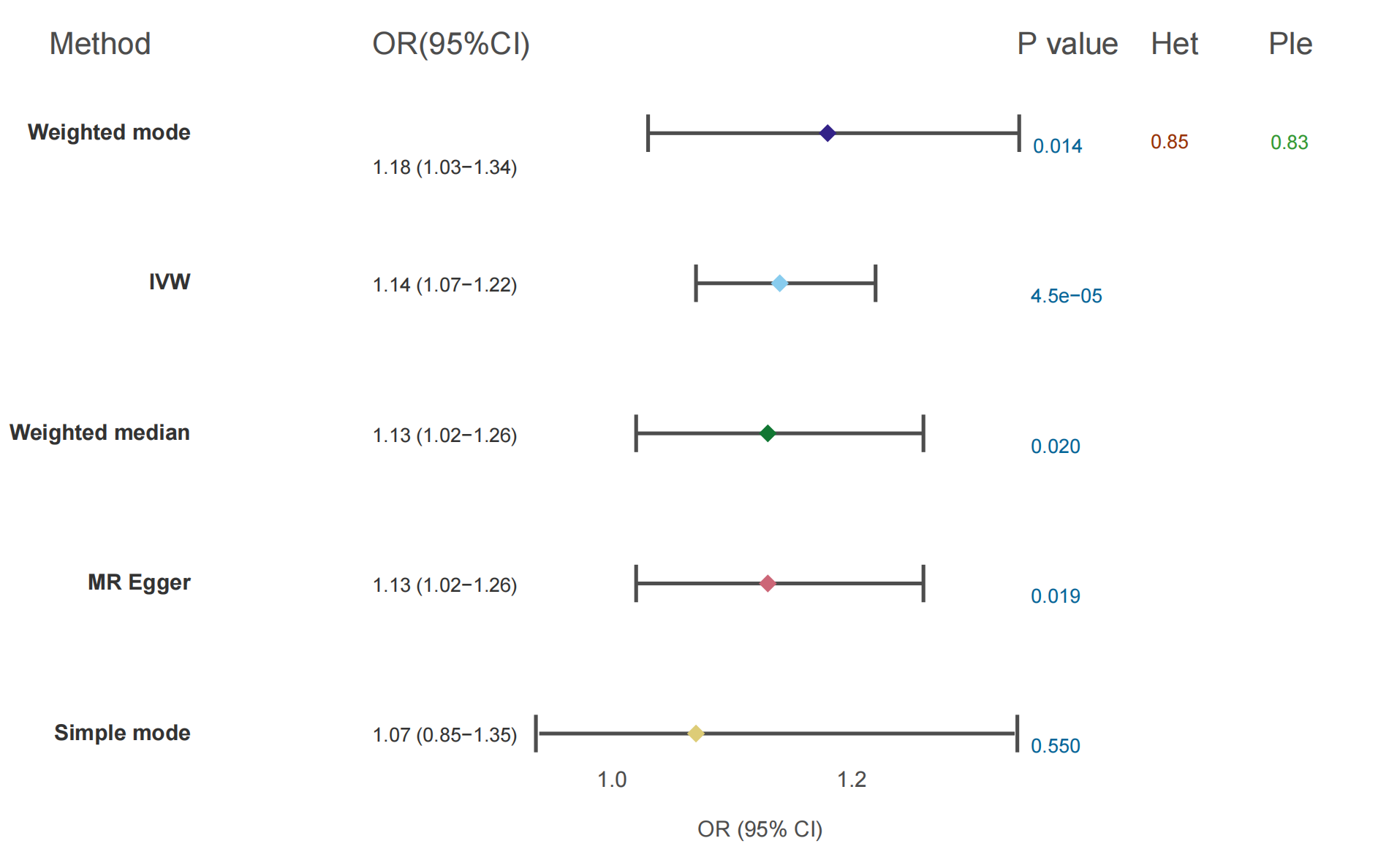
**

**Figure S7**

The forest plot displays the effect estimates for the causal influence of monocyte cell count on the risk of *ROMO1.*

**
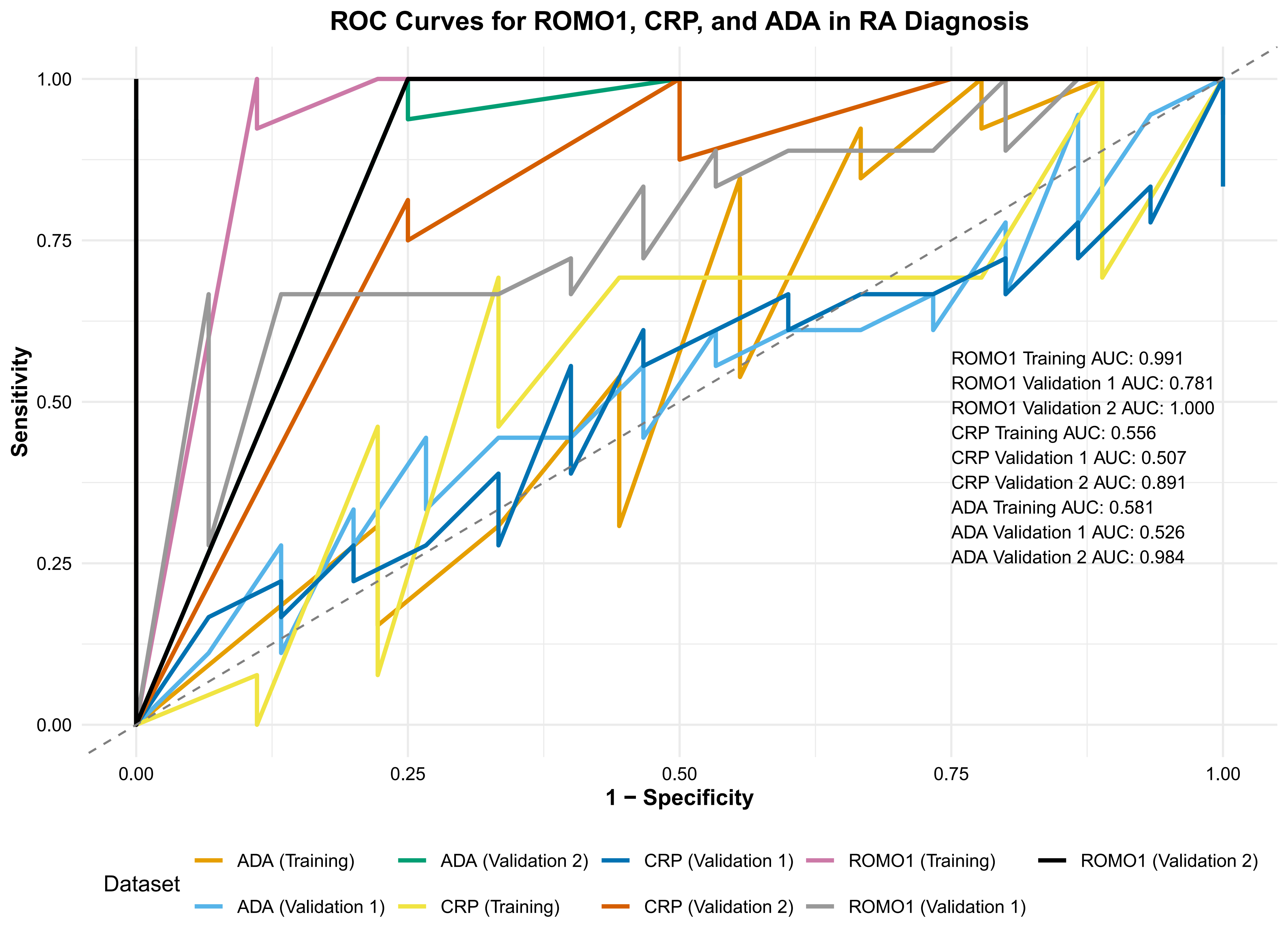
**

**Figure S8**

The ROC curves for *ROMO1, CRP,* and *ADA* in RA diagnosis.

**
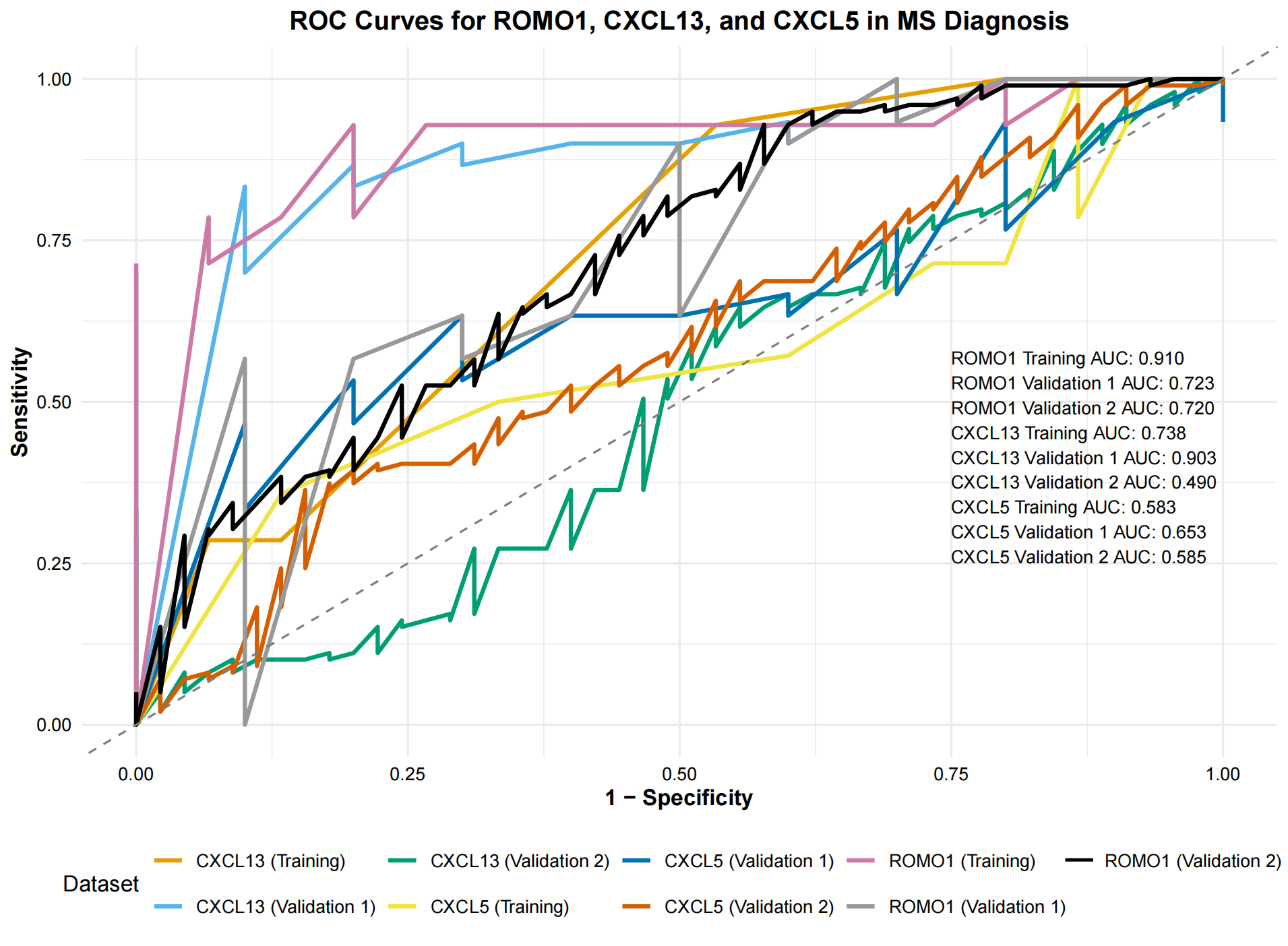
**

**Figure S9**

The ROC curves for *ROMO1, CXCL13,* and *CXCL5* in MS diagnosis.

**
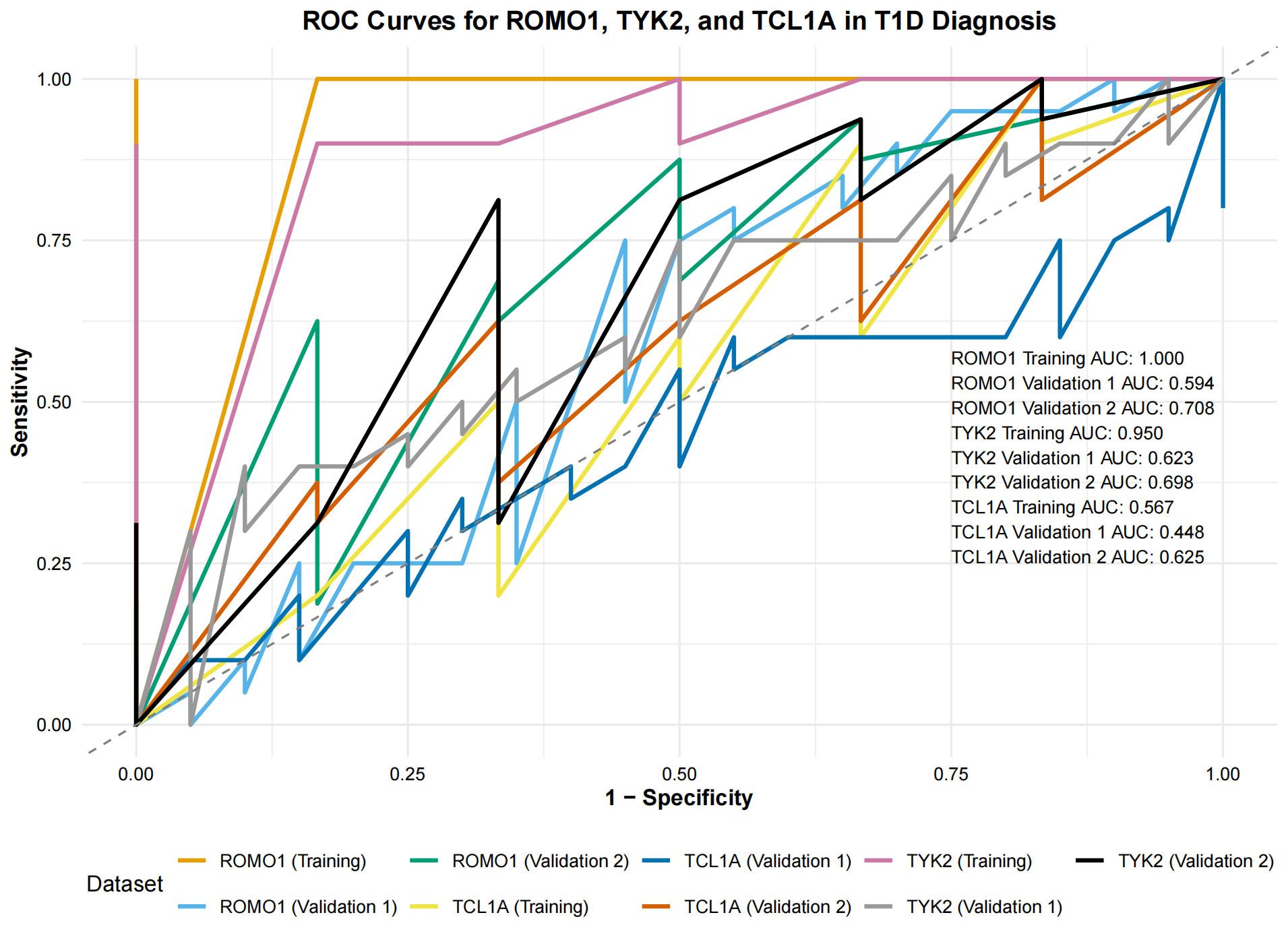
**

**Figure S10**

The ROC curves for *ROMO1, TYK2 ,* and *TCL1A* in T1D diagnosis.
