# Supplementary material for: A Multi-omics approach to identify and validate shared genetic architecture in rheumatoid arthritis, multiple sclerosis, and type 1 diabetes: integrating GWAS, GEO, MSigDB, and scRNA-seq data
Source: Funct Integr Genomics. 2025 Apr 21;25(1):91. doi: 10.1007/s10142-025-01598-x (PMC12009781; doi:10.1007/s10142-025-01598-x)
Supplement: Supplementary file 4 — Supplementary Material 4 [file 10142_2025_1598_MOESM5_ESM.docx]

**References for supplementary methods and results**

Adam, Y., Samtal, C., Brandenburg, J. T., Falola, O. & Adebiyi, E. Performing post-genome-wide association study analysis: overview, challenges and recommendations. F1000Res 10, 1002 (2021). https://doi.org:10.12688/f1000research.53962.1

Baum K, Rajapakse JC, Azuaje F. Analysis of correlation-based biomolecular networks from different omics data by fitting stochastic block models. F1000Res. 2019;8:465.

Becht, E., McInnes, L., Healy, J., Dutertre, C. A., Kwok, I. W. H., Ng, L. G., Ginhoux, F., & Newell, E. W. (2018). Dimensionality reduction for visualizing single-cell data using UMAP. Nat Biotechnol. https://doi.org/10.1038/nbt.4314

Fang S, Hemani G, Richardson TG, Gaunt TR, Davey Smith G. Evaluating and implementing block jackknife resampling Mendelian randomization to mitigate bias induced by overlapping samples. Hum Mol Genet. 2023;32(2):192-203.

Frederiksen BN, Steck AK, Kroehl M, Lamb MM, Wong R, Rewers M, et al. Evidence of stage- and age-related heterogeneity of non-HLA SNPs and risk of islet autoimmunity and type 1 diabetes: the diabetes autoimmunity study in the young. Clin Dev Immunol. 2013;2013:417657.

Itai Y, Rappoport N, Shamir R. Integration of gene expression and DNA methylation data across different experiments. Nucleic Acids Res. 2023;51(15):7762-76.

Ito, K. & Murphy, D. Application of ggplot2 to Pharmacometric Graphics. CPT Pharmacometrics Syst Pharmacol 2, e79 (2013). https://doi.org:10.1038/psp.2013.56

Jin S, Guerrero-Juarez CF, Zhang L, Chang I, Ramos R, Kuan CH, et al. Inference and analysis of cell-cell communication using CellChat. Nat Commun. 2021;12(1):1088.

Li J, Cao J, Li P, Deng R, Yao Z, Ying L, et al. A Bioinformatic Analysis of Immune-Related Prognostic Genes in Clear Cell Renal Cell Carcinoma Based on TCGA and GEO Databases. Int J Gen Med. 2022;15:325-42.

Villavicencio CN, Macrohon JJ, Inbaraj XA, Jeng JH, Hsieh JG. Development of a Machine Learning Based Web Application for Early Diagnosis of COVID-19 Based on Symptoms. Diagnostics (Basel). 2022;12(4).

Xu H, Toikumo S, Crist RC, Glogowska K, Jinwala Z, Deak JD, et al. Identifying genetic loci and phenomic associations of substance use traits: A multi-trait analysis of GWAS (MTAG) study. Addiction. 2023;118(10):1942-52.
